# Supplementary material for: Effects of global versus local trunk muscle strength training on muscle strength, proxies of power and rowing-specific performance in pubertal male rowers
Source: PLoS One. 2026 Feb 23;21(2):e0343291. doi: 10.1371/journal.pone.0343291 (PMC12928413; doi:10.1371/journal.pone.0343291)
Supplement: Attachment — Submitted filename: Point-by-point revisions-R2.pdf [file pone.0343291.s004.pdf]

Plos One
Manuscript: PONE-D-25-24075
Title: Effects of global versus local trunk muscle strength training on muscle strength, power and rowing-specific performance in pubertal male rowers
Second revision

Dear Editor,
Dear Reviewers,
We would like to express our gratitude for your valuable time and the constructive and helpful comments on our revised manuscript. We have again responded to all queries you have raised in point-by-point responses. Whenever needed, we have made changes to the re-revised second version of our manuscript. We hope that you will find the current version of our manuscript suitable for publication in Plos One.

Kind regards

Prof. Urs Granacher, PhD
On behalf of all co-authors

Reviewer #2: Changes are highlighted in yellow

The authors have substantially revised the manuscript which has greatly improved the readability and replicability of the investigation.

Author's response: We sincerely thank the reviewer for this positive feedback and for acknowledging our efforts to enhance the clarity, coherence, and methodological transparency of the manuscript. We are pleased that the revisions have improved the overall readability and replicability of our study.

Minor Issues

The authors intend on using the ST abbreviation for strength training, then once it has been defined use it consistently. Check Ln 77 and Ln 398 as examples for inconsistent use.

Author's response: We thank the reviewer for noticing this inconsistency. The abbreviation ST (strength training) has now been used consistently throughout the manuscript, including at Lines 77 and 398, as well as in all other relevant sections.

To improve the potential for the investigation outcomes to be used in future meta-analysis the authors need to report the exact p values to 3 or 4 decimal places wherever possible. See Table 1, 3,

Author's response: We appreciate the reviewer's valuable suggestion. All p values in Table 1 and Table 3, as well as throughout the manuscript, have been revised to report exact values to three decimal places, in order to enhance precision and facilitate inclusion in future meta-analyses.

Reviewer #3: Changes are highlighted in blue

GENERAL COMMENTS

The authors are commended for their substantive revisions and responsiveness to prior concerns. The manuscript demonstrates notable improvements in methodological transparency, statistical rigor, and critical interpretation of findings. However, several residual issues require clarification and refinement to achieve publication standards. The following comments delineate specific areas necessitating minor revision.

Author's response: We sincerely thank the reviewer for the positive and encouraging feedback regarding the substantial improvements made to the manuscript. We appreciate the acknowledgment of our efforts to enhance methodological transparency, statistical rigor, and interpretation of findings. We have carefully addressed all the residual issues and minor revisions outlined below to further strengthen the manuscript and ensure it meets publication standards.

Major Weaknesses

1. Methodological Confounding of Training Intensity and Modality While the authors acknowledge the confounding of exercise type (global vs. local) with loading intensity (70% 1-RM vs. bodyweight) at multiple points (Lines 283-287, 437-456, 481-485), the treatment of this limitation remains insufficient. The attempt to justify load equivalence through instability-induced muscle activation (Lines 440-451) introduces speculative reasoning that inadequately addresses the fundamental experimental design flaw. The observed superiority of GST cannot be attributed to global versus local trunk muscle recruitment patterns when external load differs substantially between conditions. This compromises the study's capacity to address its stated aim.

   Recommendation: The abstract (Lines 39-52), discussion (Lines 388-396), and conclusions (Lines 495-504) must explicitly qualify all assertions regarding GST superiority by acknowledging that effects likely reflect combined influences of exercise modality and loading intensity, not modality alone. The mechanistic interpretation attributing effects to force transmission via global musculature should be substantially tempered or eliminated.

Author's response: We thank the reviewer for this thoughtful comment highlighting the confounding between exercise modality and loading intensity. In response, we have substantially revised the abstract, discussion, and conclusion to explicitly acknowledge that the observed superiority of GST likely reflects the combined effects of exercise modality and higher external loading intensity, rather than modality alone. All mechanistic interpretations attributing these outcomes to differences in global versus local trunk muscle recruitment or force transmission have been tempered or removed. We also eliminated speculative reasoning related to instability-induced muscle

activation and clarified that the study's design limits causal inference regarding the independent effects of exercise type. These revisions ensure that all claims are appropriately qualified and accurately reflect the methodological constraints of the investigation.

2. Sample Size Justification Incongruence

Page 5, Lines 119-122: The a priori power analysis derives from bench pull strength data (Cohen's f = 0.30), yet bench pull is not assessed as a primary outcome. The appropriateness of this reference effect for estimating required sample size for trunk strength, lower-limb power, or rowing performance remains unsubstantiated. This raises concerns about whether the study was adequately powered for its stated objectives.
Recommendation: Provide explicit justification for selecting bench pull strength as the reference outcome for power estimation, or acknowledge this as a limitation affecting interpretive confidence.

Author's response: We thank the reviewer for this insightful comment. Bench pull strength was chosen as the reference outcome for the a priori power analysis because it reflects upper-body pulling capacity, which is functionally related to trunk muscle engagement during rowing, and thus serves as a reasonable proxy for expected changes in trunk strength and rowing performance. We acknowledge, however, that bench pull strength was not directly assessed as a primary outcome in the present study. This limitation was added in the end of the discussion section.

3. Hypothesis-Effect Size Discordance

Page 5, Lines 106-115: The hypothesis specifies moderate-to-large effects (Cohen's d = 0.5-0.8), yet 0.5-0.8 corresponds exclusively to "moderate" effects per Hopkins et al. [35] classification employed throughout the manuscript (small: 0.2-0.6; moderate: 0.6-1.2; large: 1.2-2.0). This inconsistency undermines precision in effect size interpretation.
Recommendation: Correct the hypothesis to state "moderate effects (d = 0.5-0.8)" or adjust to align with Hopkins criteria if large effects were genuinely anticipated.

Author's response: We thank the reviewer for highlighting this inconsistency. The hypothesis has been revised to specify moderate effects (Cohen's d = 0.5–0.8) in accordance with the Hopkins et al. [35] classification used throughout the manuscript. This correction ensures consistent and precise interpretation of the anticipated effect sizes.

Minor Weaknesses

4. Test Selection Justification (Force Plate Variables)

Page 10, Lines 244-249: The rationale for selecting jump height as the sole countermovement jump variable prioritizes "simplicity" and alignment with prior literature over biomechanical insight. Given that force plates quantify peak force, rate of force development, and power output, variables potentially more sensitive to trunk training adaptations, the exclusion of these metrics represents a missed opportunity for mechanistic understanding.

Recommendation: Strengthen justification by citing specific methodological or practical constraints that necessitated restriction to jump height, or acknowledge this as a limitation constraining mechanistic inference.

Authors response: We thank the reviewer for this important observation. We selected jump height as the primary countermovement jump variable due to methodological and practical considerations, including testing time, participant fatigue, and the feasibility of obtaining consistent measurements in a youth population as a simple, reliable, and widely used metric in youth athletes, which also facilitates comparison with previous rowing studies. We acknowledge that excluding additional force plate metrics such as peak force, rate of force development, and power output limits mechanistic insight into neuromuscular adaptations to trunk training, and we have added this in a method section and also a limitation in the manuscript as follow:

Statement included in the method section:

"Although force plates provide multiple metrics, including peak force, rate of force development, and power output, we selected jump height as the primary countermovement jump (CMJ) variable due to methodological and practical considerations. Jump height is a simple, reliable, and highly reproducible measure in youth athletes, facilitating comparisons with previous rowing research [Baker et al.; 2025, Meylan et al., 2012]. In addition, constraints related to testing time, participant fatigue, and feasibility in a young population limited our ability to include multiple force plate variables.

References used:

- Baker, J., Shillabeer, B., Brandner, C., Graham-Smith, P., Mills, P., & Read, P. (2021). Reliability, validity, and performance characteristics of elite adolescent athletes at different stages of maturity in the 10 to 5 repeated jump test. *Pediatric exercise science*, *34*(1), 20-27.
- Meylan, C. M., Cronin, J. B., Oliver, J. L., Hughes, M. G., & McMaster, D. (2012). The reliability of jump kinematics and kinetics in children of different maturity status. *The Journal of Strength & Conditioning Research*, *26*(4), 1015-1026.

Statement included in the **study limitation:**

"In addition, although force plates allow measurement of multiple variables such as peak force, rate of force development, and power output, only jump height was assessed as the primary countermovement jump outcome. This choice was made for methodological and practical reasons, including testing time, participant fatigue, and the feasibility of consistent measurements in a youth population. While jump height

is a reliable and widely used measure in youth athletes (Baker et al.; 2025, Meylan et al.; 2009), the exclusion of other force plate metrics restricts the mechanistic insight into how trunk training affects neuromuscular function and dynamic performance [**Mănescu et al., 2025**]. Future studies should incorporate these additional variables to more comprehensively evaluate training adaptations."

References used:

- Baker, J., Shillabeer, B., Brandner, C., Graham-Smith, P., Mills, P., & Read, P. (2021). Reliability, validity, and performance characteristics of elite adolescent athletes at different stages of maturity in the 10 to 5 repeated jump test. *Pediatric exercise science*, *34*(1), 20-27.
- Meylan, C. M., Cronin, J. B., Oliver, J. L., Hughes, M. G., & McMaster, D. (2012). The reliability of jump kinematics and kinetics in children of different maturity status. *The Journal of Strength & Conditioning Research*, *26*(4), 1015-1026.
- Mănescu, D. C. (2025). Computational Analysis of Neuromuscular Adaptations to Strength and Plyometric Training: An Integrated Modeling Study. Sports, 13(9), 298.

5. Inconsistent Effect Size Metrics

The manuscript alternates between Cohen's d (within-group changes), partial eta-squared (ANOVA), and unstandardized effect size ranges reported in confidence intervals. For example: Page 14, Line 333: "d = 1.22 [CI: -8.17 to 6.78]" — confidence intervals appear to represent raw score differences rather than standardized effect sizes Page 15, Line 364: Similar presentation inconsistency

Recommendation: Standardize effect size reporting throughout. If confidence intervals represent raw scores, clarify this explicitly (e.g., "CI for mean difference"). Ensure all Cohen's d values are accompanied by standardized CIs or eliminate unstandardized CIs from effect size reporting.

Authors response: We thank the reviewer for highlighting this inconsistency. We have revised the manuscript to standardize effect size reporting throughout. All Cohen's *d* values now include standardized confidence intervals, while confidence intervals for raw score differences are explicitly labeled as "CI for mean difference." Partial eta-squared values for ANOVA remain unchanged, as they are already standardized measures of effect size. These revisions ensure consistency in effect size presentation and improve interpretive clarity.

6. Redundancy and Verbosity

Page 3-4, Lines 75-93: The conceptual distinction between global and local trunk muscles is reiterated excessively

Authors response: We appreciate the reviewer's feedback. The manuscript has been revised to streamline the introduction and discussion of global and local trunk muscles, removing repetitive explanations while retaining the essential conceptual distinction needed to contextualize the study. This improves manuscript conciseness and readability without compromising clarity of the theoretical framework in the introduction section as follow:

"Even in limb-focused strength training programs, trunk muscles are recruited secondarily as force transmission structures during multi-joint exercises. While previous studies in young athletes have mainly targeted upper- and lower-limb muscles, the trunk still plays a central role in sports like rowing, transferring force from the lower limbs through a stable core to the upper limbs and ultimately to the oars to generate boat propulsion [5, 6]. Anatomically and functionally, trunk muscles can be categorized as local or global: local muscles attach segmentally to the lumbar vertebrae and primarily provide intersegmental control and spinal stability, whereas global muscles attach to the pelvis, ribcage, or long spinal lever arms, contributing to trunk torque generation, force transfer, and gross movement control [7]. Contemporary paradigms, such as the joint-by-joint training approach [8, 9], highlight the importance of targeting both local and global muscles within kinetic chains to optimize performance and reduce injury risk. Accordingly, local trunk ST (e.g., planks, bird dogs) may improve spinal stability and control, while global trunk ST (e.g., loaded trunk rotations, machine-based extensions) may enhance force production, stiffness, and athletic power."

Page 18, Lines 430-435: Repetition of GST superiority findings already established in Results
Recommendation: Condense redundant passages to enhance manuscript conciseness.

Authors response: We thank the reviewer for this observation. The manuscript has been revised to remove redundant statements regarding GST superiority in the discussion section. The revised text now emphasizes the methodological considerations and the interaction between training load and movement complexity, rather than reiterating performance differences already reported in the Results. This improves conciseness while maintaining the interpretive context of the findings in the discussion section as follow:

"The present results align with the conceptual model proposed by Hibbs et al. [12], in which global trunk training supports whole-body force production and local training contributes to postural stability and injury prevention. However, GST in this study involved externally loaded exercises (~70% 1-RM), whereas LST relied on bodyweight exercises. Therefore, the observed performance differences cannot be attributed solely to global versus local muscle recruitment, as the higher training load in GST likely contributed substantially to adaptations in strength, power, and rowing-specific performance. Although instability in LST may have increased trunk muscle activation, this remains speculative and does not resolve the confounding between exercise modality and load. Consequently, mechanistic interpretations regarding force transmission or global muscle recruitment should be made cautiously, with the findings more accurately reflecting the interaction of load magnitude and movement complexity".

SPECIFIC COMMENTS

Abstract
Line 43: "GST involved machine-based and free-weight trunk exercises, whereas LST emphasized bodyweight trunk exercises."

Issue: This phrasing obscures the critical load difference between conditions. Readers may not immediately recognize that GST utilized 70% 1-RM loading while LST relied on bodyweight.

Recommendation: Revise to: "GST involved machine-based and free-weight trunk exercises at 70% 1-RM, whereas LST emphasized bodyweight trunk exercises on stable and unstable surfaces."

Authors response: We thank the reviewer for this suggestion. The sentence has been revised to explicitly indicate the load difference: ***"GST involved machine-based and free-weight trunk exercises at approximately 70% 1-RM, whereas LST emphasized bodyweight trunk exercises on stable and unstable surfaces."*** This revision clarifies the key methodological distinction between the training conditions.

Lines 48-52: "In conclusion, GST appeared more effective than LST...however, due to the study's methodological limitations, these findings should be interpreted with caution."
Issue: The qualification is too vague. Readers require explicit identification of which methodological limitation necessitates caution.
Recommendation: Revise to: "...these findings should be interpreted with caution given that differences in external loading between conditions (70% 1-RM vs. bodyweight) confound the comparison of global versus local training modalities."

Authors response: We thank the reviewer for this suggestion. The conclusion has been revised to clarify the specific methodological limitation: *"...**these findings should be interpreted with caution given that differences in external loading between conditions (70% 1-RM vs. bodyweight) confound the comparison of global versus local training modalities."***

Introduction
Lines 106-115: Hypothesis specification

Issue: As noted in General Comment #3, the specified effect size range (0.5-0.8) corresponds to "moderate" effects per Hopkins et al., not "moderate to large."
Recommendation: Revise to: "We anticipated moderate effect sizes (Cohen's d = 0.5-0.8)..."

Authors response: We thank the reviewer for this observation. The hypothesis statement has been revised to: ***"We anticipated moderate effect sizes (Cohen's d = 0.5–0.8) favoring GST for dynamic performance measures such as countermovement jump and 700-m rowing ergometer performance."*** This revision aligns the reported

effect size range with the Hopkins et al. classification used throughout the manuscript.

Line 111: "...enhancing kinetic chain efficiency and power transfer to the limbs..." Issue: Given the methodological confounding, attributing anticipated effects specifically to kinetic chain mechanisms is speculative. Recommendation: Add qualifying language: "...potentially enhancing kinetic chain efficiency..." or defer mechanistic speculation to Discussion.

Authors response: We thank the reviewer for this comment. The sentence has been revised to include qualifying language to reflect the speculative nature of mechanistic interpretation given the confounding between training load and exercise modality. Further discussion of potential mechanisms has been moved to the Discussion section where appropriate.

Methods
Lines 151-156: Justification for omitting passive control group Issue: While ethical and practical constraints are acknowledged, the statement "previous research has already established the general effectiveness of trunk muscle ST in youth athletes [17]" requires citation verification. Reference 17 must specifically address youth athletes and trunk training effectiveness.

Recommendation: Verify that Reference 17 directly supports this claim. If not, replace with appropriate citation or remove the justification.

Authors response: We thank the reviewer for this comment. As suggested, we replace the reference 17 by the reference 39: Saeterbakken AH, Stien N, Andersen V, Scott S, Cumming KT, Behm DG, et al. The Effects of Trunk Muscle Training on Physical Fitness and Sport-Specific Performance in Young and Adult Athletes: A Systematic Review and Meta-Analysis. Sports Med. 2022;52(7):1599-622. doi: 10.1007/s40279-021-01637-0. PMID: 35061213

.
Lines 191-197: Back extensor strength test reliability Issue: This passage introduces important methodological nuance regarding test responsiveness in youth populations but reads as contradictory. First, excellent reliability (ICC = 0.98) is reported, then concerns about responsiveness are raised. Recommendation: Restructure for clarity: "While this test demonstrates excellent test-retest reliability (ICC = 0.98, SEM = 1.18%) in adult populations [20], its responsiveness to short-term training interventions in pubertal athletes may be influenced by maturational factors, motivational variability, and day-to-day performance fluctuations [21]. Observed changes should therefore be interpreted within this context."

Authors response: Revised as suggested. Thank you.

Lines 283-287: Training intensity confound

Issue: This critical limitation is buried within the training protocol description. Its significance warrants greater prominence.

Recommendation: After describing the protocols, add a separate paragraph beginning: "A notable methodological consideration is that the comparison between GST and LST is confounded by differences in external loading (70% 1-RM vs. bodyweight), which precludes definitive attribution of observed effects to muscle group targeting per se."

Authors response: Revised as suggested. Thank you.

Lines 290-301: RPE monitoring

Issue: The rationale for RPE monitoring (to address load differences) should be stated explicitly rather than implied.

Recommendation: Line 290: "To account for differences in “external” (should be “internal”) loading strategies between groups, session ratings of perceived exertion..."

Authors response: Revised as suggested. Thank you.

Results

Lines 331-334: Back extensor strength results

Issue: The confidence intervals reported [-8.17 to 6.78] appear to represent raw score differences (kg) rather than standardized effect size CIs. If so, this should be clarified. Recommendation: Clarify: "...d = 1.22 [95% CI for mean difference: -8.17 to 6.78 kg]" or report standardized CIs for Cohen's d.

Authors response: Revised as suggested. Thank you.

Similar issue: Lines 338-340, 346-349, 372-374, 378-380 — all require clarification of whether CIs represent raw or standardized metrics.

Authors response: Revised as suggested. Thank you.

Discussion
Lines 430-435: Recapitulation of results

Issue: This paragraph redundantly summarizes findings already established in Results. Discussion should prioritize interpretation, not repetition.

Recommendation: Delete Lines 430-435 and transition directly to mechanistic interpretation (Line 436 onward).

Authors response: Revised and deleted for clarity. Thank you.

Lines 437-456: Treatment of loading confound
Issue: While the authors acknowledge the confound, the subsequent appeal to instability training literature (Lines 440-451) attempts to minimize its importance by arguing that elevated muscle activation during unstable exercises may offset lower external loads. This reasoning is problematic for three reasons:
The cited instability studies (References 40-41) examined EMG activity during same-load comparisons (stable vs. unstable conditions), not comparisons between high-load stable and low-load unstable training

The argument assumes LST participants experienced muscle activation equivalent to GST despite lower loads—an assumption unsupported by direct EMG measurement in this study

Even if muscle activation were comparable, training adaptations depend on multiple factors beyond acute activation (e.g., mechanical tension, metabolic stress, training volume)
Recommendation: Substantially revise this passage. Acknowledge the limitation more directly: "The observed superiority of GST cannot be definitively attributed to global versus local muscle targeting, as the higher external loads employed in GST (70% 1-RM) likely contributed to greater strength and power adaptations independently of muscle group specificity. While unstable training may elevate muscle activation relative to stable conditions at equivalent loads [40,41], the present study did not equate external loading between conditions, nor did it assess muscle activation via electromyography. Consequently, the relative contributions of loading intensity versus exercise modality to observed adaptations remain indeterminate."

Authors response: Included as suggested. Thank you.

Lines 457-465: Rowing performance interpretation

Issue: The mechanistic explanation ("reflecting a combination of increased trunk muscle force production and higher training load") again conflates modality and intensity effects.

Recommendation: Revise to emphasize uncertainty: "...likely reflecting the combined influences of enhanced trunk strength and the higher external loading employed in GST, though their relative contributions cannot be isolated."

Authors response: Included and revised as suggested. Thank you.

Lines 481-485: Acknowledgment of loading confound in Limitations
Issue: While appropriately placed, this statement should explicitly recommend future research designs that equate loading between conditions.
Recommendation: Add: "Future investigations should compare GST and LST using load-equated protocols (e.g., progressive loading in both conditions to equivalent RPE targets) to isolate the effects of muscle group targeting from those of training intensity."

Conclusions
Lines 496-498: "This study demonstrated that six weeks of GST was more effective

than LST in improving indicators of muscle strength, power, and rowing-specific performance..."
Issue: This statement does not reflect the interpretive limitations necessitated by the methodological confound.

Recommendation: Revise to: "Six weeks of high-intensity GST produced greater improvements in muscle strength, power, and rowing-specific performance compared to bodyweight LST; however, these effects likely reflect combined influences of exercise modality and loading intensity."

Authors response: Included as suggested. Thank you.

Lines 502-504: Practical recommendations

Issue: The directive to "prioritize GST using multi-joint, free-weight exercises" overstates the evidence given the study's limitations.

Recommendation: Revise to: "Coaches may consider incorporating GST with progressive external loading as part of foundational strength development programs, while recognizing that the independent contributions of global muscle targeting versus training intensity require further investigation."

Authors response: Included and revised for clarity as suggested. Thank you.

Tables and Figures

Table 3: Effect sizes and confidence intervals

Issue: As noted previously, the confidence intervals appear to represent raw score differences rather than standardized effect size CIs. This should be clarified in the table note or the values should be standardized.

Recommendation: Add to table note: "Confidence intervals represent 95% CIs for mean differences in raw score units."

Authors response: Included in table 3 as suggested. Thank you.

References
Reference 17 (Line 590-593): Colado et al. (2011) — Verify that this citation directly supports the claim on Line 153 regarding established effectiveness of trunk ST in youth athletes. The Colado paper examines paraspinal muscle recruitment during exercises but may not specifically address training effectiveness in youth populations.

Authors response: We appreciate the reviewer's insight. Upon reviewing the Colado et al. (2011) study, we acknowledge that while it provides valuable information on paraspinal muscle recruitment during various trunk exercises, it does not directly assess the effectiveness of trunk strength training in youth populations. Therefore, we have removed this citation from Line 153.

To substantiate our claim regarding the effectiveness of trunk strength training in youth athletes, we have included the following reference:

- Saeterbakken, A.H., et al. (2022). The Effects of Trunk Muscle Training on Physical Fitness and Sport-Specific Performance in Young and Adult Athletes: A Systematic Review and Meta-Analysis. Frontiers in Physiology, 13, 850. https://doi.org/10.3389/fphys.2022.850

This systematic review and meta-analysis provides evidence supporting the effectiveness of trunk muscle training in improving various measures of physical fitness and sport-specific performance in both young and adult athletes.

We have updated the manuscript accordingly to reflect this change and ensure the accuracy of our references.
