# Supplementary material for: Effects of global versus local trunk muscle strength training on muscle strength, proxies of power and rowing-specific performance in pubertal male rowers
Source: PLoS One. 2026 Feb 23;21(2):e0343291. doi: 10.1371/journal.pone.0343291 (PMC12928413; doi:10.1371/journal.pone.0343291)
Supplement: Attachment — Submitted filename: Point-by-point revisions-R3.pdf [file pone.0343291.s005.pdf]

Plos One
Manuscript: PONE-D-25-24075
Title: Effects of global versus local trunk muscle strength training on muscle strength, power and rowing-specific performance in pubertal male rowers
Third revision

Dear Editor,
Dear Reviewers,
We would like to express our gratitude for your valuable time you have once again invested in our manuscript and the constructive and helpful comments on the revised paper. We have again responded to all of your queries in point-by-point responses. Whenever needed, we have made changes to the re-revised manuscript. We hope that you will find the current version of our manuscript suitable for publication in Plos One.

Kind regards

Prof. Urs Granacher, PhD
On behalf of all co-authors

Reviewer #2: Changes were highlighted in yellow

I acknowledge the substantial revisions the authors have undertaken and their willingness to engage with the peer review process. However, my ongoing concern is with the decision to only analyses and report on the CMJ performance purely using Jump height.

Authors' response: We thank the reviewer for acknowledging our revisions. We understand the concern regarding the exclusive use of jump height as the sole CMJ parameter. This choice was dictated by methodological constraints related to the test apparatus employed. Specifically, the Ergojump system (Globus Italia, Codognè, Italy) is an optoelectronic contact platform that measures flight time to calculate jump height but does not record force-time data (e.g., peak force, power, rate of force development). Because the device cannot provide kinetic data, we are unable to report secondary mechanical metrics. We have now clarified the functioning of the system in the methods section and expanded the limitations section to acknowledge this constraint and to recommend that future studies use equipment capable of capturing full force-time profiles using force plates for more comprehensive CMJ analyses.

Statement in the methods section (CMJ sub-section):

"The countermovement jump (CMJ) was assessed using the Ergojump system (Globus Italia, Codognè, Italy), an optoelectronic contact platform that detects take-off and landing via infrared sensors. The device calculates flight time and subsequently derives jump height using standard kinematic equations. As the system is technically not capable of recording kinetic data, i.e., force–time, peak forces,

impulses, or rate of force development we were unable to report mechanical outcomes. Therefore, jump height was used as the primary CMJ outcome parameter."

Statement in the study limitations:

"A key methodological limitation of this study is the use of the Ergojump optoelectronic platform, which provides only flight-time derived jump height and does not capture force-time characteristics. Consequently, we were unable to analyse additional mechanical variables (e.g., peak force, rate of force development, impulse) that could have offered a more comprehensive evaluation of CMJ performance. Future studies should employ equipment (i.e., force plates) capable of recording full force-time profiles to enable more detailed assessments of neuromuscular performance."

What are the methodological and practical considerations that increase the testing time, participant fatigue and feasibility burden on this population beyond already performing the 3 trials with 2 mins recovery between trials (Ln 244-252) which precludes analyzing the already performed 3 trials for alternative variables of interest (potential mechanical training induced changes)?

Authors' response: We thank the reviewer for this valuable observation. Although three CMJ trials were performed, we were unable to extract additional mechanical variables because the testing device (i.e., system Ergo jump apparatus; Globus Italia, Codogne, Italy) does not allow to assess force-time data. Therefore, this limitation stems from the technical constraints of the measurement system rather than participant burden or fatigue. We have now clarified this methodological limitation in the revised manuscript.

"Although three CMJ trials were performed, a key methodological limitation of this study is the use of the Ergojump optoelectronic system (Globus Italia, Codognè, Italy), which provides only flight-time-derived jump height and does not capture force-time characteristics. Consequently, we were unable to analyse additional mechanical variables (e.g., peak force, rate of force development, impulse) that would have offered a more comprehensive evaluation of CMJ performance. This limitation stems from the technical constraints of the equipment rather than participant burden or fatigue. Future studies should employ measurement tools such as force plates that enable full force-time profiling for more detailed assessments of neuromuscular performance."

Reviewer #3:

The authors have thoroughly and systematically addressed all the concerns raised in the previous round of review. The manuscript is substantially improved in clarity, methodological transparency, and interpretive nuance.

The most critical limitation identified previously—the methodological confounding of

training modality (Global vs. Local) with training intensity (70% 1-RM vs. bodyweight)—is now clearly and appropriately stated in the Abstract, Methods, Discussion, and Conclusion. The authors have successfully tempered their claims, shifting the interpretation from the superiority of a modality to the observed effects of a combined protocol (modality + intensity).

All minor points regarding abbreviations, p-value reporting, and justification for test selection have also been resolved.

Authors' response: We sincerely thank the reviewer for his/her positive and constructive feedback. We appreciate the recognition of our efforts to improve the clarity, methodological transparency, and interpretation of the manuscript. We are pleased that the identified methodological limitations and all remaining minor issues have now been satisfactorily addressed.
