# Supplementary material for: Effects of global versus local trunk muscle strength training on muscle strength, proxies of power and rowing-specific performance in pubertal male rowers
Source: PLoS One. 2026 Feb 23;21(2):e0343291. doi: 10.1371/journal.pone.0343291 (PMC12928413; doi:10.1371/journal.pone.0343291)
Supplement: Attachment — Submitted filename: Point-by-point revisions-R4.pdf [file pone.0343291.s006.pdf]

Plos One
Manuscript: PONE-D-25-24075
Title: Effects of global versus local trunk muscle strength training on muscle strength, power and rowing-specific performance in pubertal male rowers
Fourth revision

Dear Editor,
Dear Reviewers,
We would like to express our gratitude for your valuable time you have once again invested in our manuscript and the constructive and helpful comments on the revised paper. We have again responded to all of your queries in point-by-point responses. Whenever needed, we have made changes to the re-revised manuscript. We hope that you will find the current version of our manuscript suitable for publication in Plos One.

Kind regards

Prof. Urs Granacher, PhD
On behalf of all co-authors

Reviewer #2: Changes were highlighted in yellow

I thank the authors for their responses however their reporting of the CMJ performance data collection process needs further justification and raises questions on how rigorous was the data collection process. Specifically, in their original submission at Ln 248-249 the authors stated the use of a Kistler Quatro force plate for recording CMJ performance. During each subsequent review the authors were queried as to why only jump height was being reported as there are many more informative variables that could have been reported. The indication that a force plate was used during CMJ data collection was retained by the authors in the 1st and 2nd revised manuscript versions as the authors sought to include justifications for the approach taken. Now in this third review the authors report the use of a completely different device and process removing reference to a force plate changing to a jump timing mat, thus justifying the reporting of only jump height. But why now after 3 rounds of review has this reported change been made, with the authors not even giving the indication that it was originally in error.
Furthermore the authors have retained at Ln 252-257 their original justification related to the use of a force plate but only reporting jump height.

Clearly there is an error in the reporting and consistency of the methodology followed. Please clarify and closely proof the manuscript to ensure that the methodology reported is consistent.

Authors' response: We sincerely apologize for the inconsistency in reporting the correct and actually applied CMJ assessment device across the different manuscript versions. The mention of the Kistler Quattro force plate in the original submission was an inadvertent error that went unnoticed during the initial stages of revision. The CMJ data in this study were actually collected using a jump-timing mat (i.e., Ergojump system), which provides jump height derived from flight time as metric. No force-plate variables (e.g., peak force, rate of force development) were recorded due to the applied assessment tool. During the first two revisions, we mistakenly retained the force plate reference and attempted to justify the reporting of jump height only, which inadvertently created confusion and suggested that force plate data were available but not reported. We fully acknowledge that this was an oversight on our part.

In the third revision, we revise the paragraph as follow:

« Of note, jump height is a simple, reliable, and highly reproducible measure in young athletes and is commonly used in field-based testing, allowing for meaningful comparisons with previous research [29, 30]."

Reviewer #4: Changes are highlighted in blue

Dear Authors,

I was invited by the handling editor to participate in the peer-review process of this manuscript, which has already undergone multiple rounds of evaluation by three ad-hoc reviewers. Given that the manuscript has been revised several times and that the editor is now seeking an additional assessment, I understand that the intention is to obtain a fourth expert opinion from someone with experience in sports science, particularly in the areas of biological maturation, strength training in youth, and rowing performance. Based on my expertise in these fields, I believe I can contribute constructively to the continued improvement of your work. My comments follow below.

1. Considerations Regarding the Previous Reviews (R1, R2, and R3)

After a careful analysis of the previous reviewers' reports, it is clear that each provided valuable and complementary contributions:

• Reviewer 1 (R1) focused mainly on textual clarity, structure, standardization of abbreviations, and improvements to tables and figures. Their comments were appropriate and aimed to enhance readability and reproducibility.

• Reviewer 2 (R2) emphasized important instrumentation and methodological limitations, especially regarding the exclusive use of CMJ height, derived from flight time, which restricts deeper interpretations of mechanical power. R2 also requested exact p-values and consistency in terminology.

• Reviewer 3 (R3) raised conceptually critical points, particularly concerning the fact that the study aims to compare training modalities (global vs. local), but the groups also differ in external load (70% 1RM in GST vs. body-weight exercises in LST). This introduces a clear confounding factor, limiting causal inferences about "modality" alone.

It is evident that the authors addressed many of these critiques successfully, and the manuscript has improved. However, certain aspects especially in the statistical and analytical domain still require refinement, as discussed next.

Authors' response: We thank the handling editor for requesting an additional expert review and appreciate the reviewer's willingness to contribute their expertise. We agree with the reviewer's summary of the complementary roles played by the previous reviewers. We also acknowledge that their feedback has helped strengthen the overall quality of the manuscript. In this re-revision, we have further refined the methodological, analytical, and statistical approach to ensure clarity, accuracy, and rigor throughout the manuscript.

2. Common Points Identified by all three reviewers

The previous reviewers' comments converged on several issues:
1. Confounding between training modality and training intensity/external load Consensus that the GST vs. LST comparison is not isolated to modality.
2. Need for greater statistical transparency Including exact p-values, clearer methodological reporting, and explicit acknowledgment of assumptions.

3. Limitations of CMJ assessment — All reviewers noted the restricted interpretability when using flight-time devices.

4. Textual clarity and presentation — Recommendations for better organization, formatting consistency, and improved visual presentation of results.
These converging points reveal the central concerns that have been repeatedly highlighted throughout the review process.

Authors' response: We acknowledge these converging concerns. The confounding information between training modality and external load has been explicitly stated as a limitation, and interpretations have been adjusted accordingly. Statistical transparency has been improved through exact p-value reporting, clearer methodological descriptions, and explicit acknowledgment of analytical assumptions. We additionally provided effect sizes to illustrate the practical relevance of our outcomes. The limitations of using a flight-time-based CMJ assessment tool have been recognized in the limitations section. Finally, the manuscript has been carefully edited to improve textual clarity, formatting consistency, and the presentation of tables and figures.

3. Critical Analysis of the Statistical Procedures

The authors applied separate 2×2 ANOVAs (group × time) for each outcome, followed by paired t-tests with Bonferroni corrections. Although this is acceptable for simple designs, several limitations arise:

• Conducting multiple ANOVAs increases the risk of Type I error inflation.

• The design includes repeated measures within individuals, and traditional ANOVAs do not correctly model within-subject dependency.

• Young athletes exhibit substantial inter-individual variability, especially regarding maturation status; ANOVA does not account for this through random effects.
• ANOVAs are less robust when dealing with missing data, variance heterogeneity, and the inclusion of meaningful covariates such as maturity offset or internal training load (sRPE).
Given your research goals and the longitudinal design, a Linear Mixed Model (LMM) framework is statistically more appropriate and robust.
Recommended Model: Linear Mixed Model (LMM)
All continuous outcomes with an approximately normal distribution should be analyzed with an LMM:

Outcome ~ group * time + maturity_offset + sRPE_mean + (1 | subject)

• group (GST vs. LST) and time (pre vs. post) as fixed effects;
• optional covariates: biological maturation (maturity_offset) and sRPE;
• random intercept for each participant;
• random slope for time can be considered, though with only two time points it often yields limited benefit.

About GLMM

A Generalized Linear Mixed Model (GLMM) is only necessary for non-Gaussian outcomes (binary, counts, proportions).
Since your variables are continuous and approximately normal, a GLMM is not needed.
Why LMM is superior in this context
• Models within-subject correlations appropriately
• Incorporates covariates naturally
• Produces model-based estimated marginal means (EMMeans)
• More robust with small samples
• Accommodates heteroscedasticity and missing data
• Allows clearer interpretation of group × time effects
I strongly recommend reanalyzing all variables using LMMs and reporting EMMeans with 95% CI, along with adjusted contrasts for pre–post changes.
4. How to Implement These Analyses in JAMOVI and R
A) Using JAMOVI (GAMLj – Linear Mixed Models)
1. Load your dataset.
2. Navigate to: Analyses → Mixed Models → Linear Mixed Model (GAMLj).
3. Set:

o Dependent Variable: e.g., back_ext_strength
o Fixed Factors: group, time
o Covariates: maturity_offset, sRPE_mean
4. Under Random Effects, add:
o subject as a Random Intercept
5. Under EMMeans:
o Select group*time
o Request pairwise comparisons with corrections (Holm or Bonferroni)
6. Check Model Fit and Residual Diagnostics
7. Export EMMeans tables and plots
JAMOVI provides clean, publication-ready output with estimates, SEs, p-values, and confidence intervals.
B) Using R (lme4 + lmerTest + emmeans)
Example code:

```
library(lme4)
library(lmerTest)
library(emmeans)

model <- lmer(outcome ~ group * time + maturity_offset + sRPE_mean +
(1 | subject), data = data, REML = TRUE)

summary(model) # fixed effects
emmeans(model, ~ group * time) # adjusted means
pairs(emmeans(model, ~ group * time), adjust = "holm") # contrasts
```

For reporting:
• EMMeans (pre and post) for each group
• Difference-in-change scores (GST vs. LST) with 95% CI
• Effect sizes derived from model residual SD
• Diagnostic plots of residuals

Authors' response:

We thank the reviewer for the thoughtful and detailed statistical recommendations and for highlighting the potential advantages of linear mixed-effects models (LMMs) in longitudinal study designs. We agree that LMMs represent a powerful and flexible analytical framework, particularly for complex longitudinal datasets with multiple time points, missing observations, or non-independence structures. However, after careful consideration, we elected to retain the 2 × 2 repeated-measures ANOVA approach, with the important addition of biological maturation (maturity offset) included as a covariate, for the following reasons:

The present study employed a fully balanced design with two groups (GST vs. LST) and two fixed time points (pre- and post-test), and no missing observations. Under these conditions, repeated-measures ANOVA and LMMs yield statistically equivalent fixed-effect estimates and inferences, as documented in the methodological literature. Given the applied sports science context and the target readership, repeated-measures ANOVA provides a transparent and widely understood analytical framework. This facilitates interpretation of the primary group × time interaction without introducing additional model complexity that may not meaningfully improve inference in a two-time-point design.

In line with the reviewer's recommendation, maturity offset was included as a covariate in all analyses to account for inter-individual differences in biological maturation, which is particularly relevant in youth athlete populations. This adjustment directly addresses a key source of variability and strengthens the validity of the findings.

Furthermore, we included the following statement in both statistical procedures and study limitations as follows:

Statement in the statistical procedure:

"To account for inter-individual differences in biological maturation, maturity offset was included as a covariate in the ANOVA analyses. This approach allowed to elucidate the effects of training and time to be examined independently of maturity-related variability. Where applicable, adjusted means and interaction effects were therefore reported after accounting for PHV."

Statement in the study limitation section:

"Finally, although linear mixed models (LMM) represent a robust alternative for analyzing repeated-measures data, they were not adopted as the primary analytical approach in the present study. Instead, a two-way (2×2) ANOVA was used, consistent with the experimental design and the study objectives. While we controlled for biological maturity by accounting for age at peak height velocity (APHV), we acknowledge that the 2×2 ANOVA framework has inherent limitations, particularly its reduced flexibility in handling inter-individual variability and hierarchical data structures compared with LMM. Additionally, although the inclusion of PHV status as a covariate did not meaningfully alter the main outcomes, residual maturity-related effects cannot be entirely excluded. Therefore, caution is warranted when generalizing these findings, and future studies may benefit from employing mixed-

effects modelling approaches to further account for individual growth- and maturity-related variability."

5. Final Remarks

In conclusion, I acknowledge that the manuscript has improved substantially following previous revisions and that the authors have engaged constructively with the reviewers' comments. Nevertheless, the statistical component remains the main aspect requiring refinement. Reanalyzing the data using Linear Mixed Models will greatly enhance the robustness of your findings and allow for more accurate interpretations given the repeated-measures design and the biological variability inherent in pubertal athletes.
My recommendations aim to help strengthen the scientific rigor and clarity of your work. The topic is relevant, the design is practical and meaningful for the field, and with the suggested analytical improvements, the manuscript has clear potential to contribute significantly to research on strength training and rowing performance in

Authors' response: We sincerely thank the reviewer for his/her positive and constructive feedback. We appreciate the recognition of our efforts to improve the clarity, methodological transparency, and interpretation of the manuscript. We are pleased that the identified methodological limitations and all remaining minor issues have now been satisfactorily addressed.

Reviewer #5:

I have read the various versions of the manuscript and have verified how the improvements requested by previous reviewers have been implemented. In my opinion, the work is adequate and has great potential.
I believe this article could be referenced by others who discuss rowing as a sport.

Authors' response: We thank the reviewer for this positive evaluation. The improvements suggested by the previous reviewers have been fully implemented throughout the manuscript. We appreciate the reviewer's opinion that the work is adequate and has strong potential, and we agree that the findings may be useful as a reference for future studies discussing rowing as a sport.
