# Supplementary material for: Effects of global versus local trunk muscle strength training on muscle strength, proxies of power and rowing-specific performance in pubertal male rowers
Source: PLoS One. 2026 Feb 23;21(2):e0343291. doi: 10.1371/journal.pone.0343291 (PMC12928413; doi:10.1371/journal.pone.0343291)
Supplement: Attachment — Submitted filename: Point-by-point revisions-R5.pdf [file pone.0343291.s007.pdf]

Plos One

Manuscript: PONE-D-25-24075R5

Title: Effects of global versus local trunk muscle strength training on muscle strength, proxies of power and rowing-specific performance in pubertal male rowers

Fifth revision

Dear Editor,

Dear Reviewers,

We would like to express our gratitude for the valuable time you have once again invested in our manuscript and the constructive and helpful comments on the revised paper. We have again responded to all of your queries in point-by-point responses, especially to those of reviewer #4. Whenever needed, we have made changes to the re-revised manuscript. We hope that you will find the current version of our manuscript suitable for publication in Plos One.

Kind regards

Prof. Urs Granacher, PhD

On behalf of all co-authors

Reviewer #4: Changes were highlighted in yellow

General Overview and Recommendation

After careful evaluation of the revised manuscript and the authors' point-by-point responses to the reviewers, recommends minor revisions.

The manuscript has substantially improved across successive revision rounds and now demonstrates adequate methodological transparency, conceptual coherence, and practical relevance. The current statistical treatment is methodologically acceptable and valid given the balanced two-group, two-time-point design and the absence of missing data.

However, strongly suggests that the authors consider reanalyzing the data using a more robust statistical framework (e.g., Linear Mixed Models), which would better

accommodate inter-individual variability and maturational heterogeneity inherent to pubertal samples. If the authors decide to retain the current repeated-measures ANOVA approach, they must place stronger and more explicit emphasis on its limitations, particularly in the Study Limitations section, clearly acknowledging the inferential constraints associated with this analytical choice.

With this general recommendation stated, I'll now detail the full evaluation.

Authors' response: We thank the reviewer for the positive evaluation and for recommending minor revisions. While we acknowledge that Linear Mixed Models (LMM) would provide a more flexible framework to account for inter-individual variability and maturational heterogeneity in pubertal samples, we retained the repeated-measures ANOVA given the balanced two-group, two-time-point design and the absence of missing data and no indication of baseline between group differences. In line with the editor's recommendation, we have substantially strengthened the study limitations section, explicitly detailed the inferential constraints of this analytical choice and clarifying that the findings should be interpreted primarily at the group level.

1. Critical Analysis of the Revisions Implemented

Following a comprehensive reading of the revised manuscript and the authors' responses to all reviewers, it is evident that the authors engaged constructively and professionally with the peer-review process. The manuscript shows meaningful improvements in clarity, transparency, and interpretative caution. Nonetheless, some issues remain only partially addressed, particularly in the statistical and inferential domains.

Authors' response: We thank the reviewer for this constructive assessment and for acknowledging the improvements in clarity, transparency, and interpretative caution. In response to the remaining concerns in the statistical and inferential domains, we have further strengthened the reporting of statistical outcomes and expanded the study limitations section to more explicitly acknowledge the inferential constraints of the chosen analytical approach.

2. Convergent Points Identified Across Reviewers

A clear convergence among Reviewers 1, 2, 3, and 4 can be identified around the following central concerns. Confounding between training modality and external load All reviewers consistently noted that the comparison between global trunk strength

training (GST) and local trunk strength training (LST) is fundamentally confounded by differences in external loading (≈70% 1-RM vs. bodyweight-based exercises). Limitations of CMJ assessment methodology. The exclusive reliance on jump height derived from flight time was identified as a limitation restricting mechanistic interpretations of power-related adaptations.

Authors' response: We thank the reviewer for his/her positive comments.

Need for greater statistical transparency and rigor

Reviewers emphasized clearer reporting of exact p-values, effect sizes, and improved handling of inter-individual variability, especially in a pubertal cohort.

Authors' response: Exact p-values and effect sizes have been reported during the fourth revision of this manuscript to enhance transparency. A 2×2 repeated measures ANOVA was retained given the balanced design, two time points, and absence of missing data, and no indication of baseline between group differences. Moreover, we computed a LMM using the same data set and observed similar results as with the ANOVA approach. Accordingly, we have decided to stick with the originally selected ANOVA procedure. In accordance with the reviewer's comment, we have further highlighted this issue in the limitations section. Future studies with larger samples or additional time points should preferentially use mixed-effects modelling. All of these points have been specifically addressed in our responses below.

Textual clarity and methodological consistency

Suggestions consistently targeted improvements in structure, coherence, terminology, and methodological reporting. These converging points represent the core methodological and conceptual concerns raised throughout the review process.

Authors' response: The manuscript has been carefully revised to improve textual clarity, coherence, and consistency in terminology, as well as to ensure transparent and accurate reporting of methodological details.

3. Aspects Adequately Addressed by the Authors

Several important issues were satisfactorily resolved in the revised manuscript:

3.1 Explicit acknowledgment of the modality × load confounding

The authors now: Clearly state that the observed superiority of GST cannot be attributed solely to muscle group targeting.
Adjust the tone of the conclusions to avoid strong causal claims.

Explicitly acknowledge this limitation in both the Abstract and the Discussion, which substantially strengthens interpretative transparency.
Authors' response: We thank the reviewer for his/her affirmative comments. The tone was adjusted as suggested by the reviewer.

3.2 Correction of CMJ instrumentation inconsistencies
The authors appropriately acknowledged the initial methodological inconsistency regarding the CMJ device. The methods section is now internally coherent and aligned with the actual assessment tool used. The justification for using jump height as a valid field-based outcome in youth athletes is appropriate and well supported.
Authors' response: We thank the reviewer for his/her positive rating of our last revision.

3.3 Inclusion of biological maturation as a covariate
Accounting for maturity offset represents a meaningful methodological improvement. This adjustment enhances internal validity given the known influence of biological maturation on neuromuscular adaptations in pubertal athletes.
Authors' response: We thank the reviewer for his/her positive comment.

4. Aspects Not Fully Resolved
Despite the improvements, some limitations remain:

4.1 Retention of the traditional 2×2 repeated-measures ANOVA. While the authors justify this choice based on: a balanced design, no missing data, and only two time points, this approach still: does not optimally model inter-individual variability, lacks random effects, and is less robust in small, biologically heterogeneous samples. Although the justification is acceptable from an editorial standpoint, it does not reflect best contemporary statistical practice in pediatric sport science.
Authors' response: We agree that LMM represents best contemporary practice in pediatric sport science for capturing inter-individual variability and biological

heterogeneity. In the present study, a 2×2 repeated-measures ANOVA was retained due to the fully balanced design, absence of missing data, and no indication of baseline between group differences. To assess robustness, complementary LMM with subject-specific random intercepts were also explored and yielded results consistent and similar with the originally selected ANOVA approach. This limitation has now been acknowledged, and future studies with larger samples or additional time points should preferentially adopt mixed-effects modelling. The following paragraph was added to the section study limitations:

"Given the balanced design, the presence of only two time points, the absence of missing data, and no indication of baseline between-group differences, a 2 × 2 repeated-measures ANOVA was retained, which remains appropriate in this context despite biological heterogeneity in pediatric samples (Gueorguieva & Krystal, 2004; Baayen et al., 2008). In addition, linear mixed-effect models (LMMs) were computed using the same dataset and yielded results comparable to those obtained with the ANOVA approach. Accordingly, we elected to retain the originally selected ANOVA framework.

Nevertheless, future studies involving larger samples, additional time points, or greater developmental heterogeneity should preferentially adopt LMM to better capture individual growth trajectories, in line with contemporary recommendations in pediatric sport science (Bernards et al., 2017)."

References used:

- Gueorguieva, R., & Krystal, J. H. (2004). Move over anova: progress in analyzing repeated-measures data andits reflection in papers published in the archives of general psychiatry. *Archives of general psychiatry*, *61*(3), 310-317.
- Baayen, R. H., Davidson, D. J., & Bates, D. M. (2008). Mixed-effects modeling with crossed random effects for subjects and items. *Journal of memory and language*, *59*(4), 390-412.
- Bernards, J. R., Sato, K., Haff, G. G., & Bazyler, C. D. (2017). Current research and statistical practices in sport science and a need for change. *Sports*, *5*(4), 87.

4.2 Underutilization of internal load data (sRPE)

Although session-RPE was systematically collected: it was not incorporated as a covariate, nor explored as a mediator or moderator of training effects, representing a missed analytical opportunity.

Authors' response: We thank the reviewer for this insightful observation. sRPE was systematically collected as an internal load monitoring tool, in line with established recommendations, to verify individual training responses and ensure comparable perceived training load between groups across the intervention (Foster et al., 2001; Impellizzeri et al., 2004). Importantly, no significant between-group differences in sRPE was observed throughout the training period, indicating that internal load was well-matched between training conditions.

Given the absence of between-group differences in sRPE, a key prerequisite for its inclusion as a covariate was not met. Moreover, the study was not designed to test explanatory or mechanistic pathways linking internal load to performance adaptations. Considering the sample size, the two-time-point outcome design, and the primary inferential focus on group × time effects, incorporating sRPE as a covariate or formally testing mediation or moderation would have added model complexity without sufficient statistical power or temporal resolution to support robust inference (McLaren et al., 2018). Accordingly, sRPE was used for descriptive and control/match purposes rather than as an explanatory variable. This limitation has now been explicitly acknowledged in the study limitations section:

"Although sRPE was systematically monitored to quantify internal training load and to verify potential between-group differences (Foster et al., 2001; Impellizzeri et al., 2004), no significant differences in ratings of sRPE and no indication of baseline between group differences were observed across the intervention period. Consequently, a key prerequisite for including sRPE as a covariate was not met, and it was therefore not incorporated into the inferential statistical model nor examined as a mediator or moderator of training effects. Future studies with larger samples and multiple assessment points could integrate internal load within longitudinal or mixed-effects frameworks to better elucidate dose-response relations (McLaren et al., 2018)."

References used:

- Foster, C., Florhaug, J. A., Franklin, J., Gottschall, L., Hrovatin, L. A., Parker, S., & Dodge, C. (2001). A new approach to monitoring exercise training. *Journal of Strength & Conditioning Research*, *15*(1), 109-115.
- Impellizzeri, F. M., Rampinini, E., Coutts, A. J., Sassi, A. L. D. O., & Marcora, S. M. (2004). Use of RPE-based training load in soccer. *Medicine and science in sports and exercise*, *36*(6), 1042-1047.
- McLaren, S. J., Macpherson, T. W., Coutts, A. J., Hurst, C., Spears, I. R., & Weston, M. (2018). The relationships between internal and external measures of training load and intensity in team sports: a meta-analysis. *Sports medicine*, *48*(3), 641-658.

5. Suitability of the Manuscript for Youth Training Models

Overall, the manuscript is well aligned with contemporary youth athlete development frameworks, including:

Long-Term Athlete Development (LTAD), the Youth Physical Development Model, and tier-based athlete classification.

Positive aspects include:

age-appropriate training design, ethical and methodological consideration of youth populations, and a focus on foundational strength development. Suggested additions from a pediatric sport science perspective. To further strengthen the manuscript, Reviewer 4 recommends: Explicit discussion of trainability windows. Linking observed adaptations to proximity to PHV and heightened neuromuscular plasticity. Greater emphasis on neuromotor coordination and intersegmental control. Particularly to contextualize the functional transfer observed with GST. Expanded discussion on safety, variability, and injury prevention. Especially when comparing externally loaded exercises with instability-based training. Careful discussion of generalizability to other youth sports. This would enhance the translational impact of the findings.

Authors' response: We thank the reviewer for these valuable suggestions from a pediatric sport science perspective. The discussion section has been condensed and expanded to explicitly link the findings to the mid-PHV group, with specific reference to trainability windows, neuromuscular plasticity, neuromotor coordination, safety considerations, and cautious generalizability (Balyi et al., 2013, Lloyd & Oliver, 2012, Hall et al., 2019, Granacher et al., 2016). The following paragraph was added to the discussion section:

"Training adaptations observed in Tier 2 pubertal rowers should be interpreted within the LTAD framework which highlights heightened neuromuscular responsiveness around PHV (Lloyd & Oliver, 2012). In the present study, improvements in muscle strength and power following GST likely reflect enhanced force transmission and increased maximal force production. Accordingly, ST in general, and GST in particular may be prescribed to accommodate individual differences in growth and maturation, thereby supporting the development of movement competencies during mid-PHV (Granacher et al., 2016). Nevertheless, these findings are specific to pubertal male rowers, and caution is warranted when extrapolating the current results to other populations (e.g., female athletes) or sport disciplines (e.g., canoeing)."

References used:

- Lloyd, R. S., & Oliver, J. L. (2012). The youth physical development model: A new approach to long-term athletic development. *Strength & Conditioning Journal*, *34*(3), 61-72.
- Granacher, U., Lesinski, M., Büsch, D., Muehlbauer, T., Prieske, O., Puta, C., ... & Behm, D. G. (2016). Effects of resistance training in youth athletes on muscular fitness and athletic performance: A conceptual model for long-term athlete development. *Frontiers in physiology*, *7*, 164.

6. Evaluation of the Statistical Treatment

6.1 Adequacy of the current approach

The repeated-measures ANOVA with a maturational covariate is statistically valid. Effect size reporting is comprehensive. The absence of missing data supports the analytical choice.

Authors' response: We thank the reviewer for the positive comments.

6.2 Recommendation for more robust alternatives

Nevertheless, Linear Mixed Models (LMMs) would offer clear advantages: proper modeling of within-subject dependency, improved handling of biological variability, flexible inclusion of continuous covariates (e.g., maturity offset, sRPE), and model-based estimated marginal means with confidence intervals. The absence of

LMMs does not invalidate the findings but limits inferential robustness and alignment with current best practices in pediatric sport science.

<u>Authors' response:</u> We acknowledge that LMM are in general the preferred approach for capturing inter-individual variability in pediatric sport science. In this study, a 2×2 repeated-measures ANOVA was used due to the balanced design, absence of missing data, no indication of baseline between group differences, and only two time points, under which repeated measures-ANOVA provides valid group × time estimates. Complementary mixed-effects analyses with random intercepts produced results consistent with the ANOVA. This limitation has now been noted, and future studies with larger samples or additional time points should preferentially use mixed-effects modelling.

7. Final Assessment

The manuscript demonstrates adequate scientific quality, strong relevance, and clear improvement across revision rounds. The authors appropriately acknowledge the major methodological constraints, which reflects academic rigor and transparency. However: the modality–load confounding remains structurally unresolved, the statistical analysis could be more sophisticated, and the pediatric sport science perspective could be further expanded.

Final recommendation:

The manuscript is suitable for publication pending minor revisions, provided that the limitations of the current statistical approach are made even more explicit if alternative modeling strategies are not adopted.

<u>Authors' response:</u> We thank the reviewer for the positive evaluation of the manuscript's quality and relevance. We have further strengthened the manuscript by making the limitations related to modality-load confounding, the retained statistical approach, and the scope of the paediatric sport science perspective more explicit. In particular, the constraints associated with not adopting alternative modelling strategies are now clearly stated in the discussion and limitations sections, ensuring full transparency and alignment with best practices.

Reviewer #6: The titled "Effects of global versus local trunk muscle strength training on muscle strength, proxies of power and rowing-specific performance in pubertal male rowers" all comments have been addressed.

Authors' response: We thank the reviewer for confirming that all comments have been addressed. We appreciate the constructive feedback and are glad that the manuscript now meets the expected standards.
