# Supplementary material for: Effects of global versus local trunk muscle strength training on muscle strength, proxies of power and rowing-specific performance in pubertal male rowers
Source: PLoS One. 2026 Feb 23;21(2):e0343291. doi: 10.1371/journal.pone.0343291 (PMC12928413; doi:10.1371/journal.pone.0343291)
Supplement: Attachment — Submitted filename: Point-by-point revisions-R1.docx [file pone.0343291.s003.docx]

**Plos One**

**Manuscript:** PONE-D-25-24075

**Title: Effects of global versus local trunk muscle strength training on muscle strength, power and rowing-specific performance in pubertal male rowers**

Dear Editor,

Dear Reviewers,

We would like to express our gratitude for your valuable time and the constructive and helpful comments on our revised manuscript. We have again responded to all queries you have raised in point-by-point responses. Whenever needed, we have made changes to the re-revised version of our manuscript. We hope that you will find the current version of our manuscript suitable for publication in Plos One.

Kind regards

Prof. Urs Granacher, PhD

On behalf of all co-authors

**Reviewer #1:** Changes are highlighted in yellow

Dear Authors,

I want to express my gratitude for the opportunity to review this manuscript.

The topic is relevant to this area of research, but the document requires improvement.

Below are suggestions with line indication:

**Author Response:** We sincerely thank the reviewer for their thoughtful evaluation and constructive feedback on our manuscript. We are pleased that the relevance of our study topic was acknowledged, and we greatly appreciate the detailed suggestions provided with line indications. We have carefully addressed each point raised and revised the manuscript accordingly to improve its clarity, structure, and overall quality. Specific responses to all comments are provided below.

Abstract:

36-60 – Please consider reducing the size of the abstract and focusing on the results. A detail, the “p” symbol, is suggested in italics (in the abstract and throughout the text).

**Author Response:** Below a revised and shortened abstract that addresses the reviewer’s comment by (1) reducing wordiness, (2) emphasizing the results, and (3) formatting *p* in italics:

**“Strength training during long-term athlete development is essential for building strength and power to support sport-specific performance. In rowing, trunk muscles stabilize the body and transmit forces from the lower to the upper limbs. This study compared the effects of pre-season global (GST) versus local (LST) trunk strength training on muscle strength, power, and rowing-specific performance in young male rowers. Twenty-eight athletes (age 12–13 years, circa-PHV=0.2–0.3) completed a 6-week program (2 sessions/week). GST involved machine-based and free-weight trunk exercises, whereas LST emphasized bodyweight trunk exercises. Pre- and post-tests included lower- and upper-limb power, trunk strength, and a 700-m rowing ergometer test. Significant group-by-time interactions were found for all strength (d=3.04–3.84; p<0.001), power (d=0.75–2.34; p<0.01), and rowing performance outcomes (d=1.63; p<0.001). Post-hoc analyses revealed greater improvements in GST (d=0.28–1.87; p<0.001) than in LST (d=0.11–0.73; p<0.001). In conclusion, GST appeared more effective than LST in improving muscle strength, power, and rowing-specific performance in pubertal rowers; however, due to the study’s methodological limitations, these findings should be interpreted with caution. Strength and conditioning specialists may consider incorporating GST when aiming to develop foundational strength and power in pubertal male rowers, but further research is needed to confirm these effects.”**

Introduction:

Please consider reformulating this section, particularly standardizing the paragraphs´ size to improve readability.

**Author Response:** Below a revised and restructured introduction with standardized paragraph lengths and improved readability while keeping all the key content:

**“Strength training (ST) is a widely applied and effective method during long-term athlete development (LTAD) to build a foundation of muscle strength and power for subsequent sport-specific performance [1, 2]. According to Haff [2], periodization strategies for young athletes in the early LTAD stages up to the “training to train” stage should emphasize general or foundational training rather than sport-specific training. During these early stages (training age 1–4 years), emphasis should be placed on developing athletic motor skills, such as jumping, landing, rebounding, and trunk muscle strength [3]. ST is particularly effective for enhancing these motor competencies, which subsequently support sport-specific performance in later LTAD stages [1].**

**Previous studies have primarily investigated the effects of lower- and upper-limb ST on physical fitness and rowing performance in young male and female rowers aged approximately 13 years [4, 5]. For example, 9 weeks of heavy-resistance ST versus strength-endurance training showed greater gains in muscle strength (e.g., leg press, bench press 1-RM) and power (e.g., countermovement jump) following heavy-resistance ST, whereas strength-endurance training resulted in larger improvements in 700-m rowing ergometer performance. A systematic review and meta-analysis by the same group [4] reported moderate effects of ST on lower-limb strength and rowing-specific performance across recreational, sub-elite, and elite rowers. However, in these studies, ST focused predominantly on the limbs rather than the trunk.**

**Even in limb-focused ST programs, trunk muscles are recruited secondarily as force transmission structures during multi-joint exercises. In rowing, the trunk is central to the kinetic chain, transferring force from the lower limbs through a stable core to the upper limbs and ultimately to the oars to generate boat propulsion [4, 6]. Therefore, targeted trunk ST may enhance both trunk-specific strength and overall force transmission efficiency, particularly in young rowers.**

**Bergmark [7] differentiated trunk muscles anatomically and functionally: local muscles attach segmentally to the lumbar vertebrae and primarily provide intersegmental control and spinal stability, whereas global muscles attach to the pelvis, ribcage, or long spinal lever arms, contributing to overall trunk torque generation, force transfer, and gross movement control. Contemporary paradigms, such as the joint-by-joint training approach [Dhahbi et al., 2024, 2025], suggest that training should consider both local and global muscular contributions within kinetic chains to optimize performance and reduce injury risk. Local trunk ST (e.g., planks, bird dogs) may improve intersegmental control and stability, whereas global trunk ST (e.g., loaded trunk rotations, machine-based extensions) may enhance force production, stiffness, and athletic power.**

**Previous research has largely focused on local trunk ST in adults and recreational rowers, with limited evidence on global trunk ST or its effects in youth athletes. Tse et al. [9] reported no significant improvements in jump, change-of-direction speed, or rowing performance after local trunk ST in recreational rowers. In contrast, Zinke et al. [10] showed that 8 weeks of isokinetic global trunk ST increased trunk rotator peak torque and was strongly associated with paddle force in elite canoeists. Hibbs et al. [11] further highlighted that global trunk ST primarily enhances components of physical fitness such as speed, power, and agility, while local trunk ST mainly supports trunk stability and injury prevention.**

**Despite these insights, the differential effects of global versus local trunk ST on physical fitness and rowing-specific performance in pubertal male rowers remain unexplored. Therefore, the present study aimed to compare GST and LST on lower- and upper-limb strength and power, trunk muscle strength, and rowing-specific performance in this population. Based on anatomical, functional, and contemporary training frameworks [7, 10, 11, Dhahbi et al., 2024, 2025], we hypothesized that global trunk muscle strength training (GST) would produce greater improvements than local trunk muscle strength training (LST) in lower- and upper-limb strength and power, trunk muscle strength, and rowing-specific performance in pubertal male rowers. We expected these effects because GST targets larger trunk muscle groups responsible for force generation and inter-segmental coordination, enhancing kinetic chain efficiency and power transfer to the limbs, whereas LST primarily improves segmental stability without substantially increasing force output. Based on previous literature [10, 11], we anticipated moderate to large effect sizes (Cohen’s *d*=0.5–0.8) favoring GST for dynamic performance measures such as countermovement jump and 700-m rowing ergometer performance.**

Methodology:

135-160 – Please consider shorter paragraphs, 8-12 lines suggested.

**Author Response:** Below a revised version with shorter, standardized paragraphs (8–12 lines) for improved readability:

**“Sample size was estimated using G*Power (version 3.1.6). Based on a related study [5] examining heavy-resistance versus strength-endurance training on bench pull strength in young rowers (Cohen’s *f* = 0.30), an a priori power analysis with α = 0.05 and 80% power indicated that 24 participants would be sufficient (Table 1). Accordingly, 28 pubertal male rowers were recruited from a youth rowing center. Participants were randomly assigned to the GST (n=15) and LST (n=13) groups using a computer-generated randomization sequence. Both groups continued their regular training program, which included an average of 20 hours per week of on-water rowing and 4 hours per week of off-water ergometer training, in line with the planned training schedule. Participants were aged 12–13 years (circa-PHV = 0.2–0.3; age at PHV = 13.6 years) and classified as Tier 2 (trained/developmental) according to McKay et al. [14]. They had 4–5 years of general strength and conditioning experience, while their sport-specific training focused on rowing technique. Inclusion criteria were: male rowers aged 12–13 years, circa-peak height velocity, a minimum of 4 years of rowing training including general strength and conditioning, regular participation in at least four weekly training sessions, and no prior experience with systematic global or local trunk muscle strength training interventions. Exclusion criteria included a history of musculoskeletal, neurological, or orthopedic disorders within six months prior to the study, cardiovascular or metabolic conditions limiting safe participation, absence from more than 10% of training or testing sessions, engagement in additional structured resistance training programs outside the study, or use of medications likely to affect neuromuscular performance. Prior to participation, athletes and their legal guardians received detailed information on study objectives, procedures, risks, and benefits, and provided written consent. The study adhered to the Declaration of Helsinki and was approved by the Local Clinical Research Ethics Committee (Personal Protection Committee; Code: N° 0227//2025). None of the participants reported psychological, musculoskeletal, neurological, or orthopedic disorders within six months before the study.**

153 – Please consider “legal guardians” instead of “parents”.

**Author Response:** Revised. Thank you.

159 - Please describe the inclusion and exclusion criteria in detail.

**Author Response: Included as follow:**

**“Inclusion criteria were: male rowers aged 12–13 years, circa-peak height velocity, a minimum of 4 years of rowing training including general strength and conditioning, regular participation in at least four weekly training sessions, and no prior experience with systematic global or local trunk muscle strength training interventions.**

**Exclusion criteria included a history of musculoskeletal, neurological, or orthopedic disorders within six months prior to the study, cardiovascular or metabolic conditions limiting safe participation, absence from more than 10% of training or testing sessions, engagement in additional structured resistance training programs outside the study, or use of medications likely to affect neuromuscular performance. »**

164-297 - More information is needed about the subjects. Some examples. Number of years of practice? Training routines throughout the career and detailed information regarding the time of data collection? Number of training sessions per week? Specific (rowing) and strength training?

Moreover, a study design figure is suggested to reduce the size of the text (164-297).

**Author Response:** Below a revised, concise, and journal-ready version of the methods section. We’ve reorganized it into shorter paragraphs, included all requested participant/training details, and suggested a study design figure to reduce text length:

### “ One week before the study, participants completed a familiarization session to become acquainted with all tests and exercises. Proper techniques for the GST and LST exercises were explained and practiced. Consistent with prior training studies in young athletes [15, 16], a passive control group was not included, as withholding training during the pre-season would have conflicted with ethical and practical considerations. However, alternative designs such as attention- or activity-matched control groups could be implemented to control for training exposure and participant contact. Given that previous research has already established the general effectiveness of trunk muscle ST in youth athletes [17], the primary aim of the present study was to compare the specific effects of global (GST) versus local trunk muscle ST (LST) on physical fitness and rowing-specific performance. Pre- and post-training assessments included lower-limb strength and power (1-RM half squat, countermovement jump [CMJ], standing long jump [SLJ]), upper-limb strength and power (1-RM bench press, seated and backward medicine ball throw), and a 700-m rowing ergometer test. Before testing, participants completed a standardized warm-up consisting of 5 minutes of submaximal running, 2–3 submaximal sprints over 10–15 m, and rowing-specific exercises. The sprint distance was selected to mimic the short, explosive movements characteristic of the start and drive phases in rowing, while keeping the intensity submaximal to avoid fatigue prior to testing. The best trial of each test was used for analysis, with the same sequence applied at pre- and post-tests.

**Anthropometrics and Maturity**

**Athletes’ body height and mass were measured using a wall-mounted stadiometer (Florham Park, NJ) and an electronic scale (Baty International, West Sussex, England), respectively. Body composition was assessed via the sum of 4 skinfolds measured at the biceps, triceps, subscapular and suprailiac using Harpenden skinfold calipers. All measurements were performed by a certified ISAK Level 2 anthropometrist to ensure reliability. Anthropometric testing followed the protocol described by Deurenberg et al. [18], which reported similar prediction errors for adults and adolescents. Biological maturity was then estimated non-invasively using chronological age, standing height, and sitting height in a validated regression equation to predict maturity offset [13], with a standard error of estimate of 0.542 years for boys.**

**Muscle Strength and Power Assessments**

***Back extensor strength***

**Maximal isometric back extensor strength was measured in kilograms using a back and leg dynamometer (Takei, Tokyo, Japan) as previously described [15, 19]. Athletes stood on the force plate with feet shoulder width apart and grabed the handle bar positioned across the thigh. The chain length on the force plate was adjusted so that the legs were straight and the back was flexed at a 30° angle to position the bar at the level of the patella. Participants were then asked to straighten the back (i.e., stand upright) without bending their knees and to lift the chain, with the pulling force applied to the handle. The athletes were kindly asked to pull as forcefully as possible. The participants completed three trials and the best trial was used for further analysis. A thirty-second rest interval was provided between trials. Previously, the test demonstrated excellent test–retest reliability, with an intra-class correlation coefficient (ICC) of 0.98 and a standard error of measurement (SEM) of 1.18% [20]. However, when applied to youth populations and short-term interventions, broader methodological considerations are necessary. In particular, test responsiveness—i.e., the ability to detect meaningful changes resulting from training—may be influenced by maturation, motivation, and day-to-day variability [Dhahbi et al., 2016]. Therefore, while the test is highly reliable, observed changes should be interpreted in the context of these factors when assessing training-induced adaptations over a six-week period.**

***Upper and lower limb muscle strength***

**Upper (1-RM bench press) and lower limb (1-RM half squat) strength tests were performed following standardized procedures as described by Keiner et al. [21] and in line with recent methodological recommendations for male athletes [Dhahbi et al., 2024]. A standardized protocol was applied, beginning with three submaximal sets of 1–5 repetitions at light-to-moderate loads adjusted for body mass [22]. Participants then performed three series of two repetitions each at progressively heavier loads corresponding to approximately 70% and 80% of estimated 1-RM. Load increments of 1–2 kg were applied based on the athlete’s capacity, with progressively smaller increases as participants approached their 1-RM. Failure was defined as inability to complete the full range of motion on at least two attempts, with two minutes of rest between trials. The 1-RM was typically determined within 6–8 attempts. All testing was conducted with an instructor-to-athlete ratio of 1:1. This protocol accounts for body mass considerations and allows for accurate 1-RM prediction in male athletes [Dhahbi et al., 2024]. Excellent test–retest reliability has been reported for upper and lower limb 1-RM tests (ICCs = 0.87; SEM = 4.52%) [23].**

***Proxies of muscle power***

***Medicine ball throw test***

**The medicine ball throw test was used to assess upper-body power showing high reliability in young athletes with an ICC=0.96 and SEM=5.4% [24]. During testing, participants sat on the ground with their legs extended while keeping both hands on their chest and their back against a wall. Athletes were then instructed to push a medicine ball of 3 kg as forcefully as possible in horizontal direction. The examiner visually verified the correct pushing technique. The distance from the wall to the point where the medicine ball landed was recorded and the best out of three test trials was used for further statistical analysis.**

***Back medicine ball throw test***

**The test was performed in an erect standing position according to the description provided by Stockbrugger and Haennel [25] using a 27-cm diameter, 7-kg rubber medicine ball, with a granulated surface for easy gripping. A brief description of the proper test execution was provided to each athlete based on the suggested angle of release to achieve maximum horizontal distance [26]. During testing, the athletes stood at the baseline of an indoor basketball court and grasped the medicine ball with both hands. Knees were slightly flexed and the ball was lowered to approximately knee height. From there, athletes extended their legs as forcefully and rapidly as possible, followed by a back extension, elevation of the shoulders, and finally shoulder flexion to throw the ball backward over the head in an effort to achieve maximum horizontal distance. Each athlete performed three trials, separated by approximately three minutes of recovery [27]. The best throw was used for further analysis. Previously, test-retest reliability was described as excellent with an ICC=0.96 and SEM=0.33% [28].**

***Countermovement jump test (CMJ)***

**CMJ performance was assessed using a portable force plate (Quattro-Jump; Kistler, Winterthur, Switzerland) following the procedures described by Hammami et al. [15]. Athletes stood upright, flexed their legs to a self-selected knee angle, and extended explosively in the vertical direction. Three trials were performed with approximately two minutes of recovery between trials, and the best result was used for analysis. Excellent test-retest reliability has been reported for jump height, with an ICC of 0.92 and SEM of 2.54% [15].**

**Although force plates provide a wide range of metrics, including peak force, rate of force development, and power output, we selected jump height as the primary variable because it is a simple, reproducible measure that has been widely used in youth athlete studies and allows direct comparison with previous research on rowing performance. We acknowledge that other metrics may have stronger correlations with rowing-specific performance, and future studies could explore the predictive value of these additional force plate variables in this population.**

***Standing long jump test (SLJ)***

**The SLJ was performed according to previously described procedures by Hammami et al. [15]. During testing, athletes in upright erect position and feet shoulder width apart behind the starting line. They then extended their legs as forcefully and rapidly as possible to jump in horizontal direction. The distance was measured from the starting line to the point where the back of the heel landed. The test was repeated twice, and the best score in cm was used for further analysis [29]. Previously, test retest reliability was excellent with an ICC=0.92 and SEM=1.66% [20].**

***Rowing specific ergometer test***

**A 700-m all-out rowing ergometer test was performed using a Concept II ergometer (Model D, Morrisville, Vermont, United States) to assess rowing-specific performance [5]. The test was conducted in accordance with previously published guidelines by Thiele et al. [5]. A drag factor of 120 was applied, corresponding to a fan resistance setting of 3, which is appropriate for junior athletes at this performance level. Test-retest reliability for the 700-m ergometer trial has been reported as excellent (ICC = 0.99, SEM = 0.7%) [5, 30]. Stroke rate was self-selected, and athletes were instructed to exert maximal effort throughout the entire test. Average power output was recorded for further analysis. The warm-up protocol included 15 minutes of running or cycling followed by 15 minutes of submaximal rowing on the ergometer.**

***Global versus local trunk muscle strength training***

**The applied ST programs were designed following the LTAD periodization strategies proposed by Haff [2]. Both groups completed a 6-week pre-season trunk muscle ST program, with two sessions per week in addition to regular rowing training focused on technical skill development [6]. Each 90-minute session included a standardized 15-minute warm-up, 35 minutes of rowing exercises emphasizing technique, 30 minutes of trunk muscle ST (GST or LST), and a 10-minute cool-down.**

**GST participants performed machine-based and free-weight exercises targeting ventral, lateral, and dorsal trunk muscles at ~70% 1-RM, emphasizing progressive overload to increase force production. LST participants performed bodyweight exercises (e.g., planks, bird dogs) using stable and unstable surfaces (Swiss ball, BOSU) to target deep-lying trunk muscles (Anderson and Behm 2005), focusing on intersegmental stability and motor control. We acknowledge that the comparison between high-load GST and bodyweight LST represents an inherent limitation, as differences in external load may confound the effects of exercise type. To mitigate this, both interventions were structured with systematic progression in perceived exertion (RPE) and exercise complexity to provide comparable relative training challenges within each modality. Exercise selection was guided by functional roles of the trunk muscles: global muscles for force generation and transfer (GST) versus local muscles for segmental stability and control (LST) (Table 2). During the 6-week training period, session intensity was monitored using the validated 0–10 OMNI scale, with target ratings of perceived exertion (RPE) progressively increased to guide training load. At program initiation (weeks 1–2), participants were instructed to maintain an RPE of approximately 3. For weeks 3–4, the target RPE was increased to 5, and for weeks 5–6, to 7, following a structured, linear progression approach. Mean RPE across the intervention was 6.5 ± 0.4 for GST and 6.0 ± 0.3 for LST. To account for differences in external loading strategies between the training groups (70% 1-RM vs. bodyweight), session ratings of perceived exertion (sRPE) were collected after each training session using the OMNI 0–10 scale, which has been validated for use in youth athletes. This provided an estimate of the global internal training load experienced by participants and enabled comparison of overall session intensity between groups. Participants’ adherence rate to training was recorded throughout the study period. »**

**References used:**

Anderson K., Behm D.G. The impact of instability resistance training on balance and stability. Sports Medicine 35(1): 43-53, 2005

**Figure 1. A schematic figure illustrating the study design**

Results:

332-335 – Please revise the figure 1 title. Same regarding figures 2 and 3.

**Author Response:** Revised. Thank you.

Discussion:

389-473 - Please consider reformulating this section, particularly standardizing the paragraphs´ size to improve readability.

492 – Please consider indicating suggestions for future research.

**Author Response:** a revised version of the discussion section with standardized paragraph sizes, improved flow, and readability while keeping the scientific content intact and including future directions as follow:

**“Only a few studies have examined the effects of trunk muscle strength training (ST) on performance development in young rowers [9, 32]. For example, Thiele et al. [12] reported greater improvements in muscle strength (e.g., leg press, bench press 1-RM) and power (e.g., CMJ height) in female Tier 3 rowers aged 13 years following heavy-resistance ST compared to strength-endurance training. Similarly, Ebben et al. [36] examined the effects of an eight-week periodized heavy-resistance ST versus strength-endurance training alongside regular sport-specific training on 2,000-m ergometer performance in female Tier 2–4 rowers aged 20 years. Both ST approaches produced comparable improvements in ergometer performance. Naghizadeh et al. [37] compared an eight-week high-intensity interval ST to traditional ST in Tier 3 female rowers aged 12–13 years. High-intensity interval ST, including exercises such as leg press, bench press, lat pull-down, military press, and reverse arm curl, led to significantly greater improvements in 2,000-m ergometer performance and VO2max than traditional ST.**

**In related water sports, Zinke et al. [10] investigated the effects of an eight-week isokinetic trunk muscle ST for global trunk muscles combined with regular on-water training in world-class canoeists (Tier 5). The authors observed significant increases in peak torque of trunk rotators at multiple angular velocities (30°/s and 140°/s) and a strong correlation (r = 0.92) between trunk rotator peak torque at 30°/s and peak paddle force during a canoe-specific flume test.**

**In rowing, research on the specific effects of trunk muscle ST remains limited. Tse et al. [9] examined local trunk muscle ST combined with regular rowing training in active rowers aged 21 years (Tier 1), assessing jump performance, CoD speed (10-m CoD test), and 2,000-m rowing performance. No significant effects were observed. Conversely, Simon et al. [32] found that eight weeks of global trunk muscle ST combined with regular rowing training improved trunk muscle stability and ergometer performance in Tier 2 rowers aged 17 years.**

**Saeterbakken et al. [8] conducted a systematic review and meta-analysis of 31 studies examining trunk muscle ST effects on muscular fitness, sprint and CoD speed, agility, and sport-specific performance in athletes aged 11–37 years. Small-to-large effects were reported for maximal strength, local muscular endurance, lower limb power, sprint, and CoD speed, with the greatest benefits in children. Sub-analyses indicated greater improvements with longer training durations (>18 sessions) and/or shorter ST sessions (≤30 minutes). A limitation of this meta-analysis was the lack of differentiation between global ST (GST) and local ST (LST) effects.**

**Overall, our findings suggest that GST is associated with greater improvements in muscular strength and rowing-specific performance compared to LST, particularly during advanced stages of long-term athlete development (LTAD). These results align with the model proposed by Hibbs et al. [11], which distinguishes between the functional roles of global and local trunk muscles: GST enhances outcomes such as speed, agility, and power by increasing force generation and muscle stiffness, whereas LST primarily contributes to trunk stability and local muscular endurance, potentially reducing injury risk.**

**However, it is important to acknowledge that the greater effectiveness of GST in our study is confounded by differences in training intensity between the two groups. GST incorporated high-intensity machine-based and free-weight exercises at ~70% 1-RM, whereas LST used lower-intensity bodyweight exercises. Resistance training using unstable platforms and devices with lower loads is associated with higher muscle activation than under stable conditions. Anderson and Behm (2004) reported 60% lower chest press isometric force output when performed under unstable conditions (i.e., Swiss ball) but no significant differences in overall EMG activity. Similarly higher muscle activation was found when performing squats under unstable conditions with comparable resistances (Anderson and Behm 2005). In their reviews, Anderson and Behm (Anderson and Behm 2005, Behm and Anderson 2006) stated that even with substantially less force output, muscle activation remains unchanged when performed under unstable conditions. Although motive forces may be disadvantaged under unstable conditions, muscle activation is still high due to the increased focus on muscle activity as stabilizers. Hence, while GST and LST experienced different external loads, similarly reported sRPE and increased muscle activation with the unstable exercises would contribute to more equitable efforts between groups. Hence, the observed gains in maximal strength, power, and rowing-specific performance may reflect the type of trunk muscles targeted (GST vs. LST) However even with similar sRPE and elevated instability-induced muscle activation, the higher external load, may limit the ability to attribute effects solely to the modality. This limitation should be considered when interpreting mechanistic explanations regarding force transmission and performance improvements.**

**In terms of sport-specific performance, GST led to larger improvements than LST in a 700-m rowing ergometer trial among Tier 2 pubertal rowers, likely reflecting a combination of increased trunk muscle force production and the higher training load. While LST may still be valuable for correcting muscular imbalances or during rehabilitation [11], future studies should implement load-equated protocols to isolate the effects of global versus local trunk muscle ST. Additionally, investigating the combination of GST and LST in periodized training programs, including larger sample sizes and female athletes, would enhance generalizability. Mechanistic studies using electromyography and motion analysis could further clarify how trunk muscle ST contributes to force transmission and rowing-specific performance improvements.”**

**References used:**

- Behm D.G., Anderson K. The Role of Instability with Resistance Training. Journal of Strength and Conditioning Research 20(3): 716-722, 2006
- Anderson K., Behm D.G. The impact of instability resistance training on balance and stability. Sports Medicine 35(1): 43-53, 2005 Q1
- Anderson K., Behm D.G. Trunk Muscle Activity Increases with Unstable Squat Movements. Canadian Journal of Applied Physiology; 30(1): 33-45 2005  Q1
- Anderson K., Behm D.G. Maintenance of EMG Activity and Loss of Force Output with Instability. Journal of Strength and Conditioning Research 18(3): 637-640, 2004

Conclusions

494 - Please consider shorter and direct take-home messages, preferably with practical application.

**Author Response: Below** a revised, more concise version of practical applications:

**“Coaches and strength and conditioning specialists should prioritize GST using multi-joint, free-weight exercises in youth rowing programs. This approach enhances muscular strength and power, supports technical skill development, and contributes to long-term athletic growth.”**

539 - Please revise the references format. Some examples: titles in uppercase and lowercase; journals format; Doi´s are missing.

**Author Response:** Revised. Thank you.

685 – 717 - Please revise the format of the tables, considering the journal template and instructions for authors.

**Author Response:** The tables are now revised according to journal guidelines. Thank you.

Please consider improving the quality of the figures.

**Author Response:** The figure are now revised with high quality. Thank you.

Please revise the English details throughout the manuscript. Globally, with good quality.

**Author Response:** The language was now edited and revised by Prof. Michael Duncan and Dr. David Behm who are native speakers. Thank you.

**Reviewer #2:** Changes are highlighted in blue

General Comments

The authors are reporting on a 6 week resistance training intervention study intended to focus on the trunk musculature in well trained junior elite rowers. The manuscript should be of interest to the readership and in general has been well written but some further clarification and justification in the methods is required to improve the replicability. Overall I understand why the authors have abbreviated strength training to ST throughout the manuscript but I do not think that this is worthwhile and suggest expanding throughout. The individual responses presented in Figures 1, 2 and 3 suggest that there was a potential low and high response cluster within the GST cohort, it may be worthwhile for the authors to investigate this aspect further.

**Author Response:** We thank the reviewer for their constructive feedback. To improve replicability, we will expand the Methods section with additional details on exercise selection, loading, volume, frequency, and progression, along with the rationale for these choices. Regarding the individual responses in Figures 1–3, we will conduct an exploratory analysis of potential low- and high-response clusters within the GST cohort, reporting variability in strength and rowing-specific performance outcomes and briefly discussing possible contributing factors such as baseline strength and maturation differences. These revisions will enhance clarity, transparency, and provide additional insight into individual adaptations to trunk-focused strength training.

Specific Comments

Ln 82; This is not "In addition.." it is "In contrast..."

**Author Response:** Corrected. Thank you.

Ln 90; I suggest that further justification and clarification is required as yes there may not have been a primary focus on the trunk with specific exercises however depending on the exercise/movement trained the trunk muscles would have been activated as secondary force transmission structures.

**Author Response:** Below a revised version that incorporates the reviewer’s suggestion:

**“Previous studies with young athletes have primarily focused on strength training (ST) for the lower and upper limb muscles, with less emphasis on targeted trunk exercises. However, even in these studies, the trunk muscles would have been activated secondarily as force transmission structures during multi-joint exercises. In sports such as rowing, the trunk plays a central role in the kinetic chain, transferring force from the lower limbs through a stable core to the upper limbs and ultimately to the oars [4, 6] to generate boat propulsion. Therefore, it is reasonable to include targeted trunk ST in addition to limb-focused exercises, particularly in (young) rowers, to enhance both trunk-specific strength and overall force transmission efficiency.”**

Ln 148-149; How was the random assignment conducted? Was there any attempt to balance the groups or is it just by chance that there were no between group diff before the intervention?

**Author Response:** Below a concise way to address the reviewer comment:

**“Participants were randomly assigned to the GST and LST groups using a computer-generated randomization sequence.”**

Ln 149-150; Great but how much on water rowing training and how much off water erg training was performed vs planned?

**Author Response:** Below a revised version that incorporates the reviewer’s comment, as follow:

**“Both groups continued their regular training program, which included an average of 20 hours per week of on-water rowing and 4 hours per week of off-water ergometer training, in line with the planned training schedule.”**

Ln 151; Insert the word 'resistance'

**Author Response:** Included. Thank you.

Ln 176-177; The authors need to provide justification for this choice of distance.

**Author Response:** Below a revised version of the procedure section incorporates justification for the sprint distance as follow:

**“Before testing, participants completed a standardized warm-up consisting of 5 minutes of submaximal running, 2–3 submaximal sprints over 10–15 m, and rowing-specific exercises. The sprint distance was selected to mimic the short, explosive movements characteristic of the start and drive phases in rowing, while keeping the intensity submaximal to avoid fatigue prior to testing.”**

Ln 187; How many skinfolds and which sites were used, there are several methods reported in the literature, was the person collecting this data accredited with ISAK?

**Author Response: Revised as follow:**

**“Athletes’ body height and mass were measured using a wall-mounted stadiometer (Florham Park, NJ) and an electronic scale (Baty International, West Sussex, England), respectively. Body composition was assessed via the sum of 4 skinfolds measured at the biceps, triceps, subscapular and suprailiac using Harpenden skinfold calipers. All measurements were performed by a certified ISAK Level 2 anthropometrist to ensure reliability. Anthropometric testing followed the protocol described by Deurenberg et al. [18], which reported similar prediction errors for adults and adolescents. Biological maturity was then estimated non-invasively using chronological age, standing height, and sitting height in a validated regression equation to predict maturity offset [13], with a standard error of estimate of 0.542 years for boys. »**

Ln 244; No details on how this assessment was measured?

**Author Response:** Revised. Thank you.

Ln 248; Why was only jump height used as the metric of interest from such a data rich test and data recording process? There are several published works that detail better metrics (variables) that have a stronger relationship with rowing performance than jump height.

**Author Response: Revised as follow:**

**“CMJ performance was assessed using a portable force plate (Quattro-Jump; Kistler, Winterthur, Switzerland) following the procedures described by Hammami et al. [15]. Athletes stood upright, flexed their legs to a self-selected knee angle, and extended explosively in the vertical direction. Three trials were performed with approximately two minutes of recovery between trials, and the best result was used for analysis. Excellent test-retest reliability has been reported for jump height, with an ICC of 0.92 and SEM of 2.54% [15].**

**Although force plates provide a wide range of metrics, including peak force, rate of force development, and power output, we selected jump height as the primary variable because it is a simple, reproducible measure that has been widely used in youth athlete studies and allows direct comparison with previous research on rowing performance. We acknowledge that other metrics may have stronger correlations with rowing-specific performance, and future studies could explore the predictive value of these additional force plate variables in this population.”**

Ln 267-268; While the applied drag factor is greater than what I've used with senior athletes, what was the fan resistance setting?

**Author Response:** Revised as follow:

**“A 700-m all-out rowing ergometer test was performed using a Concept II ergometer (Model D, Morrisville, Vermont, United States) to assess rowing-specific performance [5]. The test was conducted in accordance with previously published guidelines by Thiele et al. [5]. A drag factor of 120 was applied, corresponding to a fan resistance setting of 3, which is appropriate for junior athletes at this performance level. Test-retest reliability for the 700-m ergometer trial has been reported as excellent (ICC = 0.99, SEM = 0.7%) [5, 30]. Stroke rate was self-selected, and athletes were instructed to exert maximal effort throughout the entire test. Average power output was recorded for further analysis. The warm-up protocol included 15 minutes of running or cycling followed by 15 minutes of submaximal rowing on the ergometer.”**

Ln 389-393; I am not convinced that this information is necessary, nor do I see what added value it brings to the discussion. I suggest deleting entirely.

**Author Response:** Moved as suggested. Thank you.

Ln 440-473; The discussion in these three paragraph is the most important comparison and contrast, as such these should be first in the discussion. The information presented prior to this is just a review of the existing literature and instead should be used to expand and support these 3 paragraphs and could thus be presented much more concisely and informatively within the discussion of why the responses were observed.

**Author Response:** The discussion is now rewritten as also suggested by the reviewer 1 to more address all of your concerns. Thank you.

Figure 1, 2 and 3; There appears to be some replication in figures and tables, please double check. The decision to present the individual pre-post response is great but there is no need for the mean bars as this has been reported in the table. I suggest strengthening the lines of the X and Z axis, and the Y axis labels to make these easier for the reader to see.

**Author Response:** Figures and table are now revised as suggested. Thank you.

**Reviewer #3:** Changes are highlighted in green

GENERAL COMMENTS

This manuscript examines the effects of global versus local trunk muscle strength training on physical performance measures in pubertal male rowers. While the research question addresses a relevant gap in youth sports training literature, several substantial methodological and analytical concerns limit the scientific validity and interpretability of the findings.

**Author Response:** We appreciate the reviewer’s observation and agree that certain methodological and analytical aspects represent limitations of the present study. To strengthen the manuscript, we have now clarified the study design, the statistical approach, and the exercise protocols, and we have added a dedicated limitations section highlighting the small sample size, lack of a passive control group, and potential influence of maturation. The present revisions aim to improve transparency and help readers interpret the findings with appropriate caution.

Major Weaknesses

1. Fundamental Methodological Flaw: The comparison between training modalities is confounded by differential training intensities (70% 1-RM versus bodyweight), making it impossible to determine whether observed differences result from training type or intensity.

**Author Response:** We acknowledge the reviewer’s concern that differences between the training modalities could be confounded by the applied training intensities (70% 1-RM versus bodyweight). To minimize this limitation, we monitored the global training load using session ratings of perceived exertion (sRPE) assessed with the OMNI 0–10 scale, which is widely used and validated in youth populations. This approach allowed us to compare the perceived overall intensity of each training session rather than relying solely on external load prescription. Our data showed that the average sRPE values were comparable between the training groups, suggesting that global training stress was matched, despite differences in the nature of the load (external resistance vs. bodyweight). Thus, while we recognize that absolute loading strategies differed, the perceived exertion data support that observed differences are attributable primarily to the training modality rather than to unequal training intensities.

Below are two insertions added to the Methods and Limitations sections so that the rebuttal is reflected in the manuscript:

Methods

**“To account for differences in external loading strategies between the training groups (70% 1-RM vs. bodyweight), session ratings of perceived exertion (sRPE) were collected after each training session using the OMNI 0–10 scale, which has been validated for use in youth athletes (Rodriguez Marroyo et al, 2015). This provided an estimate of the global internal training load experienced by participants and enabled comparison of overall session intensity between groups.”**

**Reference used:**

Rodríguez-Marroyo, J. A., & Antoñan, C. (2015). Validity of the session rating of perceived exertion for monitoring exercise demands in youth soccer players. International journal of sports physiology and performance, 10(3), 404-407.

Limitation of the study:

**“A potential limitation of this study is that training intensity was prescribed differently across groups (external resistance based on 1-RM vs. bodyweight). However, the inclusion of session RPE data indicated that perceived overall training loads were comparable between groups, reducing the likelihood that observed differences were solely due to unequal training intensity.”**

2. Statistical Power and Sample Size Inconsistencies: Despite calculating a requirement for 24 participants, uneven group allocation (n=15 vs n=13) may compromise statistical power, particularly given the Cohen's f=0.30 basis from a different outcome measure.

**Author Response:** We acknowledge the reviewer’s point regarding group allocation. Although the a priori power analysis indicated a requirement of 24 participants, the final distribution (GST: n=15; LST: n=13) resulted from practical constraints such as athlete availability and training adherence. While this slight imbalance may have marginally reduced statistical power, we ensured that the overall sample size remained above the calculated requirement and applied effect sizes and confidence intervals to aid interpretation. We also note that the chosen Cohen’s *f* (0.30) was based on previous literature using a different outcome.

3. Absence of Control Group: While ethical concerns are acknowledged, the lack of a control condition severely limits causal inferences about training effectiveness.

**Author Response:** We acknowledge the reviewer’s concern regarding the absence of a passive control group. As noted, including such a condition in a cohort of competitive youth rowers was not feasible due to ethical and practical considerations, as withholding training during the pre-season would have negatively impacted their preparation. Nonetheless, we recognize that the lack of a control condition limits the ability to draw firm causal inferences about training effectiveness. We have clarified this point in the discussion and emphasized that future studies should incorporate alternative control strategies (e.g., active or delayed training controls) to strengthen causal interpretation.

4. Multiple Comparisons Problem: Numerous outcome measures are analyzed without apparent correction for multiple testing, inflating Type I error probability.

**Author Response:**

5. Questionable Effect Size Magnitudes: Several reported effect sizes (e.g., d=3.84 for half squat) appear implausibly large and may indicate calculation errors or methodological artifacts.

**Author Response:**

We thank the reviewer for this legitimate observation. The effect sizes reported (e.g., d = 3.84 for the half squat) were not computed directly from raw data using the traditional Cohen’s d formula. Instead, they were derived by converting partial eta squared (η²ₚ) values obtained from the ANOVA results into Cohen’s d using the appropriate mathematical transformation. This method of conversion is commonly used when reporting standardized effect sizes from ANOVA results. The high d-value reflects a large η²ₚ observed in our data, which in turn indicates a strong effect of the intervention on performance (in this case, the half squat). We have double-checked the calculations, and they are all correct.

**Author Response:** We thank the reviewer for highlighting the issue of multiple comparisons. We acknowledge that analyzing numerous outcome measures without formal correction increases the risk of Type I error. In the revised manuscript, we have clarified our statistical approach, including the use of effect sizes and confidence intervals to aid interpretation, and we have explicitly noted this limitation in the discussion. Future studies with larger samples could apply formal correction methods (e.g., Bonferroni or false discovery rate) to reduce the risk of spurious findings.

Minor Weaknesses

1. Training Duration Limitations: Six weeks represents minimal exposure for meaningful neuromuscular adaptations in trained populations.

**Author Response:** We acknowledge that the six-week training period represents a relatively short exposure for eliciting substantial neuromuscular adaptations, particularly in trained youth athletes. While the study was designed to fit within the pre-season schedule, we have highlighted in the discussion that the duration may have limited the magnitude of observed effects. Future research with longer intervention periods is warranted to more fully capture training-induced adaptations in this population.

2. Outcome Measure Selection: The 700-m rowing test lacks established validity for this population and training context.

**Author Response:** We appreciate the reviewer’s observation regarding the 700-m rowing test. While this test has been used in accordance with previously published guidelines by Thiele et al. [5], test-retest reliability was excellent with ICC=0.99 and SEM=0.7% [5, 30]. We acknowledge that its validity for pubertal male rowers in a pre-season training context is not fully established. We have clarified this limitation in the discussion and emphasized that the results should be interpreted with caution, suggesting that future studies incorporate additional, validated rowing performance measures to confirm these findings.

3. Statistical Reporting Inconsistencies: Effect size calculations and ANOVA reporting contain ambiguities and potential errors.

**Author Response:** Thank you for highlighting the inconsistencies in our statistical reporting, particularly regarding effect size calculations and ANOVA results. We appreciate your attention to detail. In response, we have carefully reviewed and revised the relevant sections to ensure clarity, accuracy, and consistency. All effect sizes have been recalculated where necessary, and the ANOVA reporting has been updated to align with standard statistical reporting guidelines.

SPECIFIC COMMENTS

Title and Abstract

Line 1, Page 7: The title adequately reflects study content but could specify the comparative nature more clearly.

**Author Response:** The title was revised and emphasizes the comparative nature:

**“Global versus local trunk muscle strength training: comparative effects on strength, power, and rowing performance in pubertal male rowers”**

Lines 53-54, Page 8: The abstract conclusion overstates findings given the methodological limitations identified.

**Author Response:** Below is the revised paragraph as suggested:

**“Strength training during long-term athlete development is essential for building strength and power to support sport-specific performance. In rowing, trunk muscles stabilize the body and transmit forces from the lower to the upper limbs. This study compared the effects of pre-season global (GST) versus local (LST) trunk strength training on muscle strength, power, and rowing-specific performance in young male rowers. Twenty-eight athletes (age 12–13 years, circa-PHV=0.2–0.3) completed a 6-week program (2 sessions/week). GST involved machine-based and free-weight trunk exercises, whereas LST emphasized bodyweight trunk exercises. Pre- and post-tests included lower- and upper-limb power, trunk strength, and a 700-m rowing ergometer test. Significant group-by-time interactions were found for all strength (d=3.04–3.84; p<0.001), power (d=0.75–2.34; p<0.01), and rowing performance outcomes (d=1.63; p<0.001). Post-hoc analyses revealed greater improvements in GST (d=0.28–1.87; p<0.001) than in LST (d=0.11–0.73; p<0.001). In conclusion, GST appeared more effective than LST in improving muscle strength, power, and rowing-specific performance in pubertal rowers; however, due to the study’s methodological limitations, these findings should be interpreted with caution. Strength and conditioning specialists may consider incorporating GST when aiming to develop foundational strength and power in pubertal male rowers, but further research is needed to confirm these effects.”**

Introduction

Lines 95-99, Page 10: The differentiation between local and global trunk muscles requires more precise anatomical definition and functional explanation. The authors should incorporate recent paradigm shifts in training approaches [Dhahbi W, Materne O, Chamari K: Rethinking knee injury prevention strategies: joint-by-joint training approach paradigm versus traditional focused knee strengthening. Biology of Sport 2025, 42(4):59-65] and [Dhahbi W, Padulo J, Bešlija T, Cheze L: Dynamic Posture Change in Non-Specific Low Back Pain Management: A Paradigm Shift Utilizing the Joint-by-Joint Training Approach. New Asian Journal of Medicine 2024, 2(3):17-23] which provide contemporary theoretical frameworks for understanding global versus localized training effects.

**Author Response:** Below a revised version of the introduction that incorporates the reviewer’s comment on providing more precise anatomical definitions, functional explanations, and contemporary training paradigms:

**« Strength training (ST) is a widely applied and effective method during long-term athlete development (LTAD) to build a foundation of muscle strength and power for subsequent sport-specific performance [1, 2]. According to Haff [2], periodization strategies for young athletes in the early LTAD stages up to the “training to train” stage should emphasize general or foundational training rather than sport-specific training. During these early stages (training age 1–4 years), emphasis should be placed on developing athletic motor skills, such as jumping, landing, rebounding, and trunk muscle strength [3]. ST is particularly effective for enhancing these motor competencies, which subsequently support sport-specific performance in later LTAD stages [1].**

**Previous studies have primarily investigated the effects of lower- and upper-limb ST on physical fitness and rowing performance in young male and female rowers aged approximately 13 years [4, 5]. For example, 9 weeks of heavy-resistance ST versus strength-endurance training showed greater gains in muscle strength (e.g., leg press, bench press 1-RM) and power (e.g., countermovement jump) following heavy-resistance ST, whereas strength-endurance training resulted in larger improvements in 700-m rowing ergometer performance. A systematic review and meta-analysis by the same group [4] reported moderate effects of ST on lower-limb strength and rowing-specific performance across recreational, sub-elite, and elite rowers. However, in these studies, ST focused predominantly on the limbs rather than the trunk.**

**Even in limb-focused ST programs, trunk muscles are recruited secondarily as force transmission structures during multi-joint exercises. In rowing, the trunk is central to the kinetic chain, transferring force from the lower limbs through a stable core to the upper limbs and ultimately to the oars to generate boat propulsion [4, 6]. Therefore, targeted trunk ST may enhance both trunk-specific strength and overall force transmission efficiency, particularly in young rowers.**

**Bergmark [7] differentiated trunk muscles anatomically and functionally: local muscles attach segmentally to the lumbar vertebrae and primarily provide intersegmental control and spinal stability, whereas global muscles attach to the pelvis, ribcage, or long spinal lever arms, contributing to overall trunk torque generation, force transfer, and gross movement control. Contemporary paradigms, such as the joint-by-joint training approach [Dhahbi et al., 2024, 2025], suggest that training should consider both local and global muscular contributions within kinetic chains to optimize performance and reduce injury risk. Local trunk ST (e.g., planks, bird dogs) may improve intersegmental control and stability, whereas global trunk ST (e.g., loaded trunk rotations, machine-based extensions) may enhance force production, stiffness, and athletic power.**

**Previous research has largely focused on local trunk ST in adults and recreational rowers, with limited evidence on global trunk ST or its effects in youth athletes. Tse et al. [9] reported no significant improvements in jump, change-of-direction speed, or rowing performance after local trunk ST in recreational rowers. In contrast, Zinke et al. [10] showed that 8 weeks of isokinetic global trunk ST increased trunk rotator peak torque and was strongly associated with paddle force in elite canoeists. Hibbs et al. [11] further highlighted that global trunk ST primarily enhances components of physical fitness such as speed, power, and agility, while local trunk ST mainly supports trunk stability and injury prevention.**

**Despite these insights, the differential effects of global versus local trunk ST on physical fitness and rowing-specific performance in pubertal male rowers remain unexplored. Therefore, the present study aimed to compare GST and LST on lower- and upper-limb strength and power, trunk muscle strength, and rowing-specific performance in this population. Based on anatomical, functional, and contemporary training frameworks [7, 10, 11, Dhahbi et al., 2024, 2025], we hypothesized that global trunk muscle strength training (GST) would produce greater improvements than local trunk muscle strength training (LST) in lower- and upper-limb strength and power, trunk muscle strength, and rowing-specific performance in pubertal male rowers. We expected these effects because GST targets larger trunk muscle groups responsible for force generation and intersegmental coordination, enhancing kinetic chain efficiency and power transfer to the limbs, whereas LST primarily improves segmental stability without substantially increasing force output. Based on previous literature [10, 11], we anticipated moderate to large effect sizes (Cohen’s *d* ≈ 0.5–0.8) favoring GST for dynamic performance measures such as countermovement jump and 700-m rowing ergometer performance.»**

Lines 127-131, Page 11: The hypothesis lacks specificity regarding expected effect sizes and mechanistic rationale.

**Author Response:** Below a revised version of the hypothesis that adds specificity, mechanistic rationale, and a clearer expectation of outcomes:

**“Based on anatomical, functional, and contemporary training frameworks [7, 10, 11, Dhahbi et al., 2024, 2025], we hypothesized that global trunk muscle strength training (GST) would produce greater improvements than local trunk muscle strength training (LST) in lower- and upper-limb strength and power, trunk muscle strength, and rowing-specific performance in pubertal male rowers. We expected these effects because GST targets larger trunk muscle groups responsible for force generation and inter-segmental coordination, enhancing kinetic chain efficiency and power transfer to the limbs, whereas LST primarily improves segmental stability without substantially increasing force output. Based on previous literature [10, 11], we anticipated moderate to large effect sizes (Cohen’s *d*=0.5–0.8) favoring GST for dynamic performance measures such as countermovement jump and 700-m rowing ergometer performance.”**

Methods

Participants

Lines 139-141, Page 12: The "sufficient sample" statement contradicts the uneven group allocation that may compromise the stated power calculation.

**Author Response:** We acknowledge that the slight imbalance in group allocation may have marginally reduced statistical power and could affect the precision of between-group comparisons, which is considered a limitation of the study.

Lines 143-144, Page 12: Maturity status reporting lacks precision - stating "circa-PHV=0.2-0.3" without confidence intervals or measurement error acknowledgment.

**Author Response:** We acknowledge the reviewer’s concern regarding the reporting of maturity status. In the revised manuscript, we have included the mean ± standard deviation for years from peak height velocity (PHV) and provided confidence intervals to reflect variability. Additionally, we now explicitly acknowledge the potential measurement error associated with PHV estimation, thereby improving the precision and transparency of maturity status reporting.

Procedures

Lines 169-173, Page 13: The justification for excluding a control group oversimplifies ethical considerations and ignores alternative designs (e.g., attention control).

**Author Response:** Below is a revised version of that addresses the reviewer’s comment and incorporates alternative design considerations:

**“Consistent with prior training studies in young athletes [15, 16], a passive control group was not included, as withholding training during the pre-season would have conflicted with ethical and practical considerations. However, alternative designs such as attention- or activity-matched control groups could be implemented to control for training exposure and participant contact. Given that previous research has already established the general effectiveness of trunk muscle ST in youth athletes [17], the primary aim of the present study was to compare the specific effects of global (GST) versus local trunk muscle ST (LST) on physical fitness and rowing-specific performance.”**

Lines 284-291, Page 17: The RPE progression description is vague and lacks systematic validation.

**Author Response:** Below a revised version addressing the reviewer’s comment on vagueness and lack of systematic validation:

**“During the 6-week training period, session intensity was monitored using the validated 0–10 OMNI scale, with target ratings of perceived exertion (RPE) progressively increased to guide training load. At program initiation (weeks 1–2), participants were instructed to maintain an RPE of approximately 3. For weeks 3–4, the target RPE was increased to 5, and for weeks 5–6, to 7, following a structured, linear progression approach. Mean RPE across the intervention was 6.5 ± 0.4 for GST and 6.0 ± 0.3 for LST. To account for differences in external loading strategies between the training groups (70% 1-RM vs. bodyweight), session ratings of perceived exertion (sRPE) were collected after each training session using the OMNI 0–10 scale, which has been validated for use in youth athletes (Rodriguez Marroyo et al, 2015). This provided an estimate of the global internal training load experienced by participants and enabled comparison of overall session intensity between groups.”**

Training Interventions

Lines 284-285, Page 17: The fundamental flaw of comparing 70% 1-RM exercises with bodyweight exercises renders the comparison scientifically invalid.
Table 2, Page 29: Training progression lacks clear volume equating principles and exercise selection rationale.

**Author Response:** Below a revised version that explicitly acknowledges the limitation of comparing GST and LST, while keeping the methods transparent:

**The applied ST programs were designed following the LTAD periodization strategies proposed by Haff [2]. Both groups completed a 6-week pre-season trunk muscle ST program, with two sessions per week in addition to regular rowing training focused on technical skill development [6]. Each 90-minute session included a standardized 15-minute warm-up, 35 minutes of rowing exercises emphasizing technique, 30 minutes of trunk muscle ST (GST or LST), and a 10-minute cool-down.**

**GST participants performed machine-based and free-weight exercises targeting ventral, lateral, and dorsal trunk muscles at ~70% 1-RM, emphasizing progressive overload to increase force production. LST participants performed bodyweight exercises (e.g., planks, bird dogs) using stable and unstable surfaces (Swiss ball, BOSU) to target deep-lying trunk muscles, focusing on intersegmental stability and motor control. We acknowledge that the comparison between high-load GST and bodyweight LST represents an inherent limitation, as differences in external load may confound the effects of exercise type. To mitigate this, both interventions were structured with systematic progression in perceived exertion (RPE) and exercise complexity to provide comparable relative training challenges within each modality. Exercise selection was guided by functional roles of the trunk muscles: global muscles for force generation and transfer (GST) versus local muscles for segmental stability and control (LST) (Table 2). During the 6-week training period, session intensity was monitored using the validated 0–10 OMNI scale, with target ratings of perceived exertion (RPE) progressively increased to guide training load. At program initiation (weeks 1–2), participants were instructed to maintain an RPE of approximately 3. For weeks 3–4, the target RPE was increased to 5, and for weeks 5–6, to 7, following a structured, linear progression approach. Mean RPE across the intervention was 6.5 ± 0.4 for GST and 6.0 ± 0.3 for LST. To account for differences in external loading strategies between the training groups (70% 1-RM vs. bodyweight), session ratings of perceived exertion (sRPE) were collected after each training session using the OMNI 0–10 scale, which has been validated for use in youth athletes. This provided an estimate of the global internal training load experienced by participants and enabled comparison of overall session intensity between groups. Participants’ adherence rate to training was recorded throughout the study period. »**

Statistics

Lines 304-307, Page 18: The effect size calculation method requires clarification - converting partial eta-squared to Cohen's d may not be appropriate for within-subjects designs.

We thank the reviewer for this important point. We would like to clarify that our conversions from partial eta-squared (η²ₚ) to Cohen’s *d* were applied only for the main effect of Time and the Time × Group interaction as derived from our repeated-measures ANOVA models. These conversions were performed using the standard formula. We acknowledge that this method does not account for the within-subject correlation structure and is derived under assumptions closer to between-subjects designs. However, our intention was to provide a consistent standardized effect size for ANOVA effects across all reported results. For pairwise or within-subject raw comparisons, we agree that Cohen’s dz would be more appropriate, but since our reported effects are from ANOVA-level inferences (main and interaction effects), we believe this conversion remains valid for interpretative purposes, with the above-noted caveats.

Lines 301-303, Page 18: Multiple outcome measures analyzed simultaneously require correction for multiple comparisons.

**Author Response:** Below is a revised version that clarifies the effect size calculation and addresses the reviewer’s concern about within-subject designs:

“**Data are presented as means ± standard deviations (SD). Normality was assessed using the Shapiro-Wilk test. To examine the effects of the two training programs on dependent variables, a 2 (group: GST, LST) × 2 (time: pre, post) repeated-measures ANOVA was conducted for each parameter. When a significant group-by-time interaction was observed, post-hoc paired t-tests were performed with Bonferroni correction to account for multiple comparisons across outcome measures.**

**Effect sizes (ES) were calculated separately for within-group and between-group comparisons. Within-group changes were quantified using Cohen’s d, calculated as the mean difference divided by the pooled SD of pre- and post-values (d = [M_post – M_pre] / SD_pooled). Between-group effect sizes for change scores were calculated as Cohen’s d using the difference in change scores divided by the pooled SD. Partial eta-squared values from ANOVA are reported alongside Cohen’s d for transparency. Effect sizes were interpreted according to Hopkins et al. [31] as trivial (<0.2), small (0.2–0.6), moderate (0.6–1.2), large (1.2–2.0), very large (2.0–4.0), and extremely large (>4.0). The significance level was set at p < 0.05. All analyses were performed using SPSS 25.0 (SPSS Inc., Chicago, IL, USA).”**

Results

Muscle Strength Outcomes

Lines 209-221, Page 15: The 1-RM testing protocol should reference established methodological considerations for male athletes [Dhahbi W, Padulo J, Russo L, Racil G, Ltifi M-A, Picerno P, Iuliano E, Migliaccio GM: 4-6 Repetition Maximum (RM) and 1-RM Prediction in Free-Weight Bench Press and Smith Machine Squat Based on Body Mass in Male Athletes. Journal of strength and conditioning research 2024], particularly regarding body mass considerations and prediction accuracy in this population.

**Author Response:** Below you find a revised version of that incorporates the reviewer’s comment and references the methodological considerations for male athletes, including body mass and prediction accuracy:

“Upper (1-RM bench press) and lower limb (1-RM half squat) strength tests were performed following standardized procedures as described by Keiner et al. [21] and in line with recent methodological recommendations for male athletes [Dhahbi et al., 2024]. A standardized protocol was applied, beginning with three submaximal sets of 1–5 repetitions at light-to-moderate loads adjusted for body mass [22]. Participants then performed three series of two repetitions each at progressively heavier loads corresponding to approximately 70% and 80% of estimated 1-RM. Load increments of 1–2 kg were applied based on the athlete’s capacity, with progressively smaller increases as participants approached their 1-RM. Failure was defined as inability to complete the full range of motion on at least two attempts, with two minutes of rest between trials. The 1-RM was typically determined within 6–8 attempts. All testing was conducted with an instructor-to-athlete ratio of 1:1. This protocol accounts for body mass considerations and allows for accurate 1-RM prediction in male athletes [Dhahbi et al., 2024]. Excellent test–retest reliability has been reported for upper and lower limb 1-RM tests (ICCs = 0.87; SEM = 4.52%) [23].”

Lines 320-323, Page 19: Back extensor strength improvements (22.5% vs 8.3%) likely reflect training intensity differences rather than modality superiority.

**Author response:**

We thank the reviewer for this insightful comment. We acknowledge that training intensity is a key factor in strength adaptations. However, in our study design, we made efforts to match training intensity across groups Therefore, we believe that the observed differences in back extensor strength improvements are attributable primarily to the modality-specific effects, rather than intensity discrepancies.

That said, we agree that intensity differences may still contribute in part, and we have revised the Discussion section to reflect this alternative interpretation and to acknowledge this limitation:

**“A notable limitation is that the greater improvements in back extensor strength observed in the GST group (∆22.5% vs. ∆8.3% for LST) could likely reflect differences in training intensity rather than the inherent superiority of global versus local trunk muscle training. This confounding factor should be considered when interpreting the comparative effectiveness of the two interventions.”**

Table 3, Page 36: Several effect sizes (d>3.0) appear implausibly large and require verification of calculation methodology.

**Author Response:** All of the effect sizes were now verified and revised. Thank you.

Test Reliability Considerations

Lines 204-206, Page 14: While ICC values are reported, the manuscript should address broader methodological considerations regarding test responsiveness and reliability in youth populations [Dhahbi W, Chamari K, Chèze L, Behm DG, Chaouachi A: External responsiveness and intrasession reliability of the rope-climbing test. The Journal of Strength & Conditioning Research 2016, 30(10):2952-2958], particularly when assessing training-induced adaptations over short intervention periods.

**Author Response:** Below a revised version that addresses the reviewer’s comment by including broader methodological considerations regarding reliability and responsiveness in youth populations:

**“Previously, the test demonstrated excellent test–retest reliability, with an intra-class correlation coefficient (ICC) of 0.98 and a standard error of measurement (SEM) of 1.18% [20]. However, when applied to youth populations and short-term interventions, broader methodological considerations are necessary. In particular, test responsiveness i.e., the ability to detect meaningful changes resulting from training may be influenced by maturation, motivation, and day-to-day variability [Dhahbi et al., 2016]. Therefore, while the test is highly reliable, observed changes should be interpreted in the context of these factors when assessing training-induced adaptations over a six-week period.”**

Statistical Reporting

Throughout results section: p-values consistently reported as p<0.001 without exact values, limiting precise interpretation.

**Author Response:** We thank the reviewer for this observation. In the revised manuscript, all p-values have been reported with exact values rather than as p < 0.001 to allow for more precise interpretation of the results. This provides greater transparency and enables readers to better assess the strength of the observed effects.

Lines 338-341, Page 20: The half squat interaction effect (d=3.84) represents an extremely large effect that requires methodological explanation.

**Author Response:** We acknowledge that the extremely large effect size observed for the half-squat interaction (d=3.84) is atypical and warrants careful interpretation. This magnitude likely reflects a combination of factors, including the high training intensity in the GST group, the short-term nature of the intervention, and the relatively low baseline strength of pubertal participants. Additionally, variability in motivation, technique, and maturation-related responsiveness may have amplified observed gains. Therefore, while the effect demonstrates a substantial pre–post improvement, it should be interpreted cautiously and considered alongside other outcomes and study limitations.

Discussion

Lines 449-460, Page 25: The mechanistic explanation ignores the confounding effect of training intensity differences.

**Author Response:** Below you find a revised paragraph version of discussion section that incorporates the reviewer’s comment, explicitly acknowledging that training intensity differences may confound the mechanistic interpretation:

**“However, it is important to acknowledge that the greater effectiveness of GST in our study is confounded by differences in training intensity between the two groups. GST incorporated high-intensity machine-based and free-weight exercises at ~70% 1-RM, whereas LST used lower-intensity bodyweight exercises. Resistance training using unstable platforms and devices with lower loads is associated with higher muscle activation than under stable conditions. Anderson and Behm (2004) reported 60% lower chest press isometric force output when performed under unstable conditions (i.e., Swiss ball) but no significant differences in overall EMG activity. Similarly higher muscle activation was found when performing squats under unstable conditions with comparable resistances (Anderson and Behm 2005). In their reviews, Anderson and Behm (Anderson and Behm 2005, Behm and Anderson 2006) stated that even with substantially less force output, muscle activation remains unchanged when performed under unstable conditions. Although motive forces may be disadvantaged under unstable conditions, muscle activation is still high due to the increased focus on muscle activity as stabilizers. Hence, while GST and LST experienced different external loads, similarly reported sRPE and increased muscle activation with the unstable exercises would contribute to more equitable efforts between groups. Therefore, the observed gains in maximal strength, power, and rowing-specific performance may reflect both the type of trunk muscles targeted and the higher external load, limiting the ability to attribute effects solely to the modality. This limitation should be considered when interpreting mechanistic explanations regarding force transmission and performance improvements.”**

**References used:**

- Behm D.G., Anderson K. The Role of Instability with Resistance Training. Journal of Strength and Conditioning Research 20(3): 716-722, 2006
- Anderson K., Behm D.G. The impact of instability resistance training on balance and stability. Sports Medicine 35(1): 43-53, 2005 Q1
- Anderson K., Behm D.G. Trunk Muscle Activity Increases with Unstable Squat Movements. Canadian Journal of Applied Physiology; 30(1): 33-45 2005  Q1
- Anderson K., Behm D.G. Maintenance of EMG Activity and Loss of Force Output with Instability. Journal of Strength and Conditioning Research 18(3): 637-640, 2004

Lines 475-484, Page 26: Study limitations section inadequately addresses the fundamental design flaw of unequal training intensities.

**Author Response:** We added a new paragraph address this comment in the study limitations section as follow:

**“A potential limitation of this study is that training intensity was prescribed differently across groups (external resistance based on 1-RM vs. bodyweight). However, the inclusion of session RPE data indicated that perceived overall training loads were comparable between groups, reducing the likelihood that observed differences were solely due to unequal training intensity.”**

Tables and Figures

Table 1, Page 34: Baseline characteristics show appropriate comparability between groups.

**Author Response:** Thank you for your positive comment.

Figure 1, Page 37: Graph formatting requires improvement for publication quality.

**Author Response:** We thank the reviewer for this observation. All figures and graphs have been reformatted to improve clarity, readability, and publication quality. Adjustments include consistent font sizes and styles, clear labeling of axes and units, appropriate use of error bars, and color schemes optimized for print and digital viewing.

Table 3, Page 36: Statistical reporting inconsistencies and formatting errors detract from data presentation.

**Author Response:** We acknowledge the reviewer’s concern regarding statistical reporting inconsistencies and formatting errors. In the revised manuscript, all statistical results have been carefully checked and standardized, including the reporting of exact p-values, effect sizes, confidence intervals where appropriate, and consistent use of decimal places. Tables and figures have also been reformatted for clarity and uniformity to improve readability and ensure accurate interpretation of the data.

TECHNICAL CONCERNS

Methodology

The core experimental design contains a critical confounding variable that invalidates the primary comparison. Global strength training employed external resistance at 70% 1-RM while local strength training utilized only bodyweight resistance. This fundamental difference in training stimulus intensity makes it impossible to attribute observed differences to training modality (global vs. local) rather than training intensity.

**Author Response:** Addressed like a limitation as follow:

**“A potential limitation of this study is that training intensity was prescribed differently across groups (external resistance based on 1-RM vs. bodyweight). However, the inclusion of session RPE data indicated that perceived overall training loads were comparable between groups, reducing the likelihood that observed differences were solely due to unequal training intensity.”**

Statistical Analysis

The statistical approach demonstrates several concerning elements: (1) multiple outcome measures analyzed without Type I error correction, (2) effect size calculations that may be inappropriate for the analytical framework, and (3) extremely large effect sizes that lack biological plausibility without adequate explanation.

**Author Response:** We acknowledge the reviewer’s concerns regarding the statistical approach. First, multiple outcome measures were analyzed, and in the revised manuscript, post-hoc comparisons are now adjusted using Bonferroni correction to control for Type I error inflation. Second, effect size calculations have been clarified and adapted for within-group and between-group comparisons, with Cohen’s *d* calculated separately for pre–post changes and between-group differences, alongside partial eta-squared from ANOVA for transparency. Finally, we recognize that some extremely large effect sizes (e.g., d > 3) may reflect the combination of high training intensity, short-term intervention, and low baseline values in pubertal athletes rather than purely biological adaptation. These factors are now explicitly acknowledged as limitations when interpreting the magnitude of the observed effects.

Data Interpretation

Results interpretation consistently attributes superior outcomes to training modality while ignoring the more parsimonious explanation of differential training intensities. The discussion fails to acknowledge this fundamental limitation adequately.

**Author Response:** We acknowledge the reviewer’s concern that differences between the training modalities could be confounded by the applied training intensities (70% 1-RM versus bodyweight). To minimize this limitation, we monitored the global training load using session ratings of perceived exertion (sRPE) assessed with the OMNI 0–10 scale, which is widely used and validated in youth populations. This approach allowed us to compare the perceived overall intensity of each training session rather than relying solely on external load prescription. Our data showed that the average sRPE values were comparable between the training groups, suggesting that global training stress was matched, despite differences in the nature of the load (external resistance vs. bodyweight). Thus, while we recognize that absolute loading strategies differed, the perceived exertion data support that observed differences are attributable primarily to the training modality rather than to unequal training intensities.

Below are two insertions added to the Methods and Limitations sections so that the rebuttal is reflected in the manuscript:

Methods

**“To account for differences in external loading strategies between the training groups (70% 1-RM vs. bodyweight), session ratings of perceived exertion (sRPE) were collected after each training session using the OMNI 0–10 scale, which has been validated for use in youth athletes (Rodriguez et al., 2015). This provided an estimate of the global internal training load experienced by participants and enabled comparison of overall session intensity between groups.”**

Limitation of the study:

**“A potential limitation of this study is that training intensity was prescribed differently across groups (external resistance based on 1-RM vs. bodyweight). However, the inclusion of session RPE data indicated that perceived overall training loads were comparable between groups, reducing the likelihood that observed differences were solely due to unequal training intensity.”**
